# Runs of homozygosity reveal candidate genes for economic traits in Danish Large White pigs

**DOI:** 10.5194/aab-69-25-2026

**Published:** 2026-01-12

**Authors:** Weimin Ding, Xudong Wu, Yu Bu, Wei Zhang, Yuanlang Wang, Yueyun Ding, Xianrui Zheng, Xiaodong Zhang, Zongjun Yin

**Affiliations:** 1 Anhui Antai Agricultural Development Co., Ltd, Guangde City 242200, China; 2 Anhui Provincial Key Laboratory of Livestock and Poultry Product Safety Engineering, Institute of Animal Husbandry and Veterinary Medicine, Anhui Academy of Agricultural Sciences, Hefei City 230001, China; 3 College of Animal Science and Technology, Anhui Agricultural University, Hefei City 230031, China

## Abstract

Analysis of runs of homozygosity (ROH) in commercial breed genomes is important for accurately assessing the population inbreeding status and exploring homozygous regions related to economic traits formed by selection pressure. The Danish Large White (LW) pig is a commercially important breed renowned for its superior growth efficiency and reproductive performance. In the present study, we identified ROH segments of Danish LW pigs based on 43 individual whole-genome resequencing data. We then calculated the inbreeding coefficient and screened candidate genes with important economic traits from the ROH islands. A total of 9446 ROH segments were identified in the LW pig population. Each LW pig carried 219.67 ROH. Most ROH were <5Mb , and the average genomic inbreeding coefficient ( $F_{\text{ROH}}$ ) in LW pigs was 0.24. However, the proportion of ROH ( >5Mb ) in LW pigs has reached 10 %, indicating selection pressure or inbreeding in recent times. Candidate genes related to reproductive traits (*ALDH1A2*, *APQ9*, *ACTG1*, *CDK6*, *ADAMTS9*, *PAPPA2*, and *ESR2*), and growth and development traits (*NDN*, *CEP128*, *NFATC1*, *JAK2*, *KCNQ1*, *ANKRD22*, *ACTA2*, *FABP4*, *FAS*, *GDF15*, and *FGF21*) were identified in the genomic ROH islands of LW pigs. In conclusion, the present study provides further assessment of genetic diversity and inbreeding in the Danish LW pig population. In addition, our results provide useful insights into the functions of ROH on a hereditary basis and the role that ROH play in controlling the excellent characteristics of Danish LW pigs.

## 1 Introduction

Homozygosity, which is the occurrence of identical alleles at a genetic locus, is a crucial concept in genomics. Runs of homozygosity (ROH) are continuous stretches of homozygous genotypes in the genome, indicating that an individual inherits identical haplotypes from both parents (Purfield et al., 2012). ROH primarily occur because of inbreeding and identity by descent (Ceballos et al., 2018). Inbreeding increases the likelihood that an individual will inherit the same segment of DNA from both parents, leading to homozygosity, and the presence of ROH is often more pronounced in populations with higher rates of consanguinity or in small isolated groups with limited genetic diversity (Swinford et al., 2023). Identity by descent refers to genomic segments inherited from a common ancestor (Ji et al., 2024). Chromosomal recombination and genetic diversity reduce the likelihood of homozygosity over generations. Therefore, longer chromosomal segments shared between individuals typically indicate a recent common ancestor, whereas shorter segments suggest a more distant relationship. This provides valuable insights into the history of inbreeding and ancestral connections (Nosrati et al., 2021; Li et al., 2024). In addition, genetic drift, breeding plans, selection intensity, effective population size, population structure, and genetic linkages influence ROH development in the genome (Peripolli et al., 2017).

ROH have a wide range of applications that are crucial for gaining insights into the history of populations. These include past bottlenecks, founder effects, and demographic changes (Talebi et al., 2020). They can also be used to assess genetic diversity in populations, helping identify the extent of inbreeding and its impact on populations. In livestock, intensive artificial selection and breeding practices often lead to reduced genetic diversity and elevated levels of inbreeding. In contrast to indigenous horse breeds, those requiring studbook registration have pronounced inbreeding, as measured by elevated $F_{\text{ROH}}$ values (Chen et al., 2023). There is a significant disparity in ROH between European and Asian wild boars. This is characterized by abundant large ROH in Asian populations, which is indicative of recent demographic declines. In contrast, European wild boars have a more uniform ROH distribution, which may be attributed to ancient glacial bottlenecks and a prolonged history of a small effective population size (Tao et al., 2025).

Additionally, ROH hold potential importance for research into genetic diseases and inbreeding depression because homozygous regions increase the risk of co-expression of recessive deleterious alleles (Lynch et al., 2023). Studies on domesticated animals, including dogs, cats, and horses, have shown that selective breeding practices have increased the genetic homogeneity of certain breeds, often at the expense of genetic health, leading to higher rates of genetic diseases (Matsumoto et al., 2021; Mooney et al., 2021; Hill et al., 2022; Subramanian and Kumar, 2024). A large-scale association analysis indicated that an increase in genomic inbreeding coefficients ( $F_{\text{ROH}}$ ) causes a decrease in semen quality in Holstein bulls (Ghoreishifar et al., 2023). Lozada-Soto et al. (2024) reported that cows with a high level of genomic inbreeding have an increased likelihood of disease. Evidence of the adverse effects of ROH on economic traits has also been found in American and Canadian Duroc populations, and different breeding directions result in differences in the distribution and number of adverse genomic ROH (Wang et al., 2022).

Commercial breeds undergo strong artificial positive selection towards target traits within a short period, and purposeful homologous hybridization is beneficial for establishing these target characteristics (Pemberton et al., 2012; Ceballos et al., 2018). Therefore, the detection and functional identification of ROH hotspots can facilitate the identification of candidate genes associated with genetic gains in various economically important traits (Zhao et al., 2024). The Danish Large White (LW) pig is a valuable breed in the pork industry and is known for its growth efficiency, reproductive performance, and high-quality meat (Wu et al., 2024). In the present study, we obtained whole-genome single nucleotide polymorphism (SNP) information from 43 Danish LW pigs and identified key candidate genes associated with productive performance in the ROH islands. Our results enrich the molecular marker sites in Danish LW pigs and provide valuable insights into their characteristics.

## 2 Materials and methods

### 2.1 Samples and resequencing

In the present study, we collected 43 ear tissue samples from Danish LW pigs from Anhui Daziran Pig Breeding Co., Ltd, Huaibei City, Anhui Province, People's Republic of China. All samples were randomly selected, and the extracted DNA was subsequently sent to the Beijing Genomics Institute (Wuhan, China) for library preparation and whole-genome resequencing using the DNBSEQ platform. Sequencing was performed at a coverage depth of approximately 5× following rigorous quality control inspections (ranging from 4.88 to 6.03× per sample). Detailed descriptions of the data and sequencing methods are available in our previous study (Wu et al., 2024). In the present study, VCFtools 0.1.14 was used to remove indels (i.e., insertion–deletions) from the markers, and the PLINK v1.9 software package was used to filter the SNP sites and preserve only Danish LW autosomal SNPs (Purcell et al., 2007). High-quality SNPs with minor allele frequencies>0.05 and missing genotype rate<0.5 were kept to detect ROH. After filtering, a total of 2 021 963 high-quality SNPs remained for use in ROH detection analysis. We used the PLINK software package to calculate a genomic kinship matrix based on high-quality SNPs, with no full-sib or half-sib relationships among the individuals selected for sequencing (Table S1 in the Supplement).

### 2.2 ROH, $F_{\text{ROH}}$ , and ROH island detection

ROH identification in each LW pig was performed using PLINK v1.9 with a homozygous function. The analysis parameters were set as follows: (1) homozyg-homozyg-density, 500; (2) homozyg-gap, 1000; (3) homozyg-kb, 1000; (4) homozyg-snp, 50; (5) homozyg-window-het, 1; (6) homozyg-window-missing, 5; (7) homozyg-window-snp, 50; and (8) homozyg-window-threshold, 0.05.

The genomic inbreeding coefficient ( $F_{\text{ROH}}$ ) was calculated as the ratio of the total length of ROH fragments to the total length of the genome. We calculated $F_{\text{ROH}}$ at the chromosomal, individual, and population levels to provide a thorough understanding of the genetic structure and inbreeding patterns in the Danish LW pig population. For each SNP locus, the proportion of SNP loci in ROH regions (ROH ratio) was calculated on a population-wide basis. A Manhattan plot was constructed based on the ROH ratios of each SNP locus. The threshold for high-frequency SNPs was set at the top 1 % ROH ratio, and ROH islands were identified based on the distribution of SNP loci that exceeded this threshold across the genome.

### 2.3 Candidate gene annotation and quantitative trait loci overlap

We used the online platform OmicShare Tools (https://www.omicshare.com/tools/, last access: 2 December 2024) to perform gene ontology (GO) enrichment analysis and Kyoto Encyclopedia of Genes and Genomes (KEGG) pathway analysis to further investigate the functional roles of candidate genes located in the ROH islands (Mu et al., 2024). Significant enrichment was defined for GO terms and KEGG pathways with a Benjamini–Hochberg-corrected p value of <0.05 . Quantitative trait locus (QTL) mapping is important for understanding the genetic basis of complex traits (Liu et al., 2024a). We obtained published pig QTL information from the pig QTL database (Sscrofa 11.1; https://www.animalgenome.org/cgi-bin/QTLdb/index/, last access: 26 December 2024), overlapped it, and annotated it with the Danish LW pig ROH islands (Hu et al., 2022). Finally, we specifically focused on candidate genes that were located both in ROH islands in the present study and the selective sweep regions identified by Wu et al. (2025).

## 3 Results

**Figure 1:**
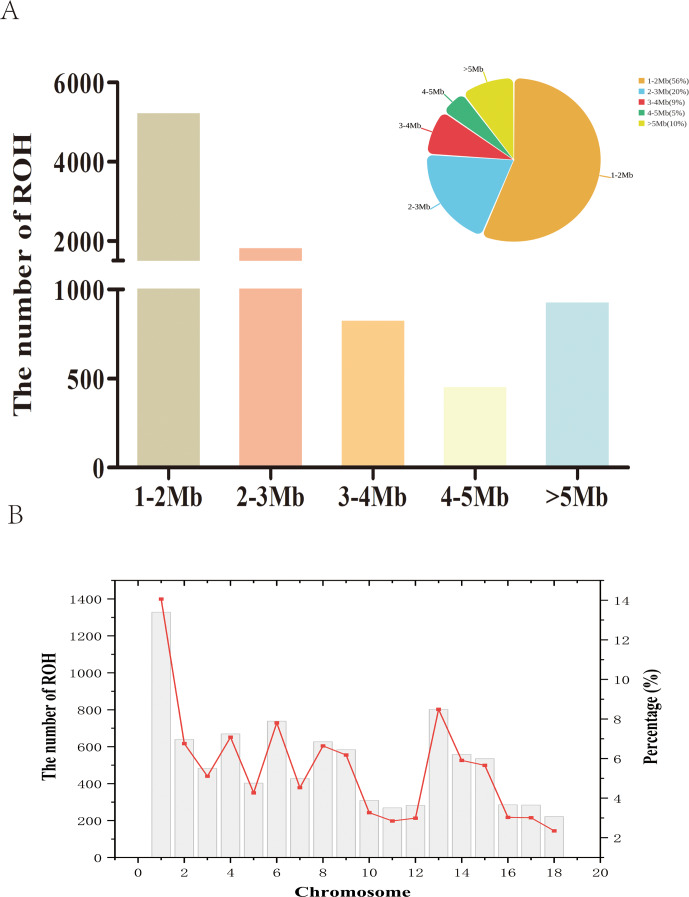
Number of ROH segments in the Danish LW pig population. **(A)** Number of ROH of different lengths. **(B)** Number of ROH in chromosomes. ROH=runs of homozygosity.

We identified 9446 ROH fragments in an LW pig population based on established screening thresholds. On average, each LW pig carried 219.67 ROH, with a median ROH length of 1.79 Mb and a mean length of 2.55 Mb (Table 1). The longest detected ROH segment was 26.08 Mb, indicating extensive homozygosity in the LW genome. As a descriptive summary of the ROH length categories (1–2, 2–3, 3–4, 4–5, >5Mb ) in Fig. 1A, the distribution of ROH lengths revealed that the majority of segments fell within the 1–2 Mb range, accounting for 5298 ROH (56 % of the total; Fig. 1A). In contrast, only 5 % of ROH were observed in the 4–5 Mb category, highlighting the predominance of shorter ROH segments in the population. At the chromosomal level, chromosome 1 harbored the highest number of ROH (1328 segments, 14 % of the total), whereas chromosome 18 exhibited the lowest count, with only 222 ROH (Fig. 1B).

Table 1 presents descriptive statistics for the genomic inbreeding coefficients ( $F_{\text{ROH}}$ ) in the LW population. Individual $F_{\text{ROH}}$ values ranged from 0.06 to 0.36, with a population-wide mean of 0.24. We also calculated the chromosome-level $F_{\text{ROH}}$ for each autosome in the LW pigs and discovered a range of 0.18–0.34 across the chromosomes. Among all autosomes, chromosome 1 exhibited the highest inbreeding coefficient, whereas chromosome 11 had the lowest values in the LW population.

**Table 1:** Descriptive statistics of ROH numbers and $F_{\text{ROH}}$ values in the LW pig population.

	Minimum	Median	Maximum	Mean	Std. dev.
Length (Mb)	1.00	1.79	26.08	2.55	2.09
$F_{\text{ROH}}$	0.06	0.26	0.36	0.24	0.09

ROH = runs of homozygosity; $F_{\text{ROH}}=\text{genomic}$ inbreeding coefficient; LW = Large White.

**Figure 2:**
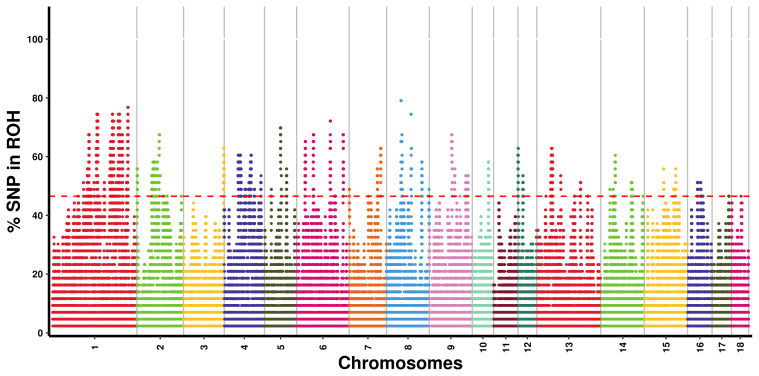
Manhattan plot of SNP incidence for ROH across individuals in the Danish LW pig population. SNP=single nucleotide polymorphism; ROH=runs of homozygosity; LW=Large White.

As illustrated in Fig. 2, we identified areas with potential biological significance by assessing the proportion of SNPs in the ROH regions of each autosome. A total of 262 ROH islands were detected across all autosomes, excluding chromosomes 11 and 18; chromosome 1 harbored the highest number of ROH islands ( n=87 ), whereas chromosome 10 contained only one island. In terms of length, the longest ROH island was observed on chromosome 4, measuring 2931.32 kb, whereas the shortest was found on chromosome 13 (3.31 kb). A total of 209 ROH islands harbored 1347 genes, whereas 53 ROH islands did not harbor any genes.

**Figure 3:**
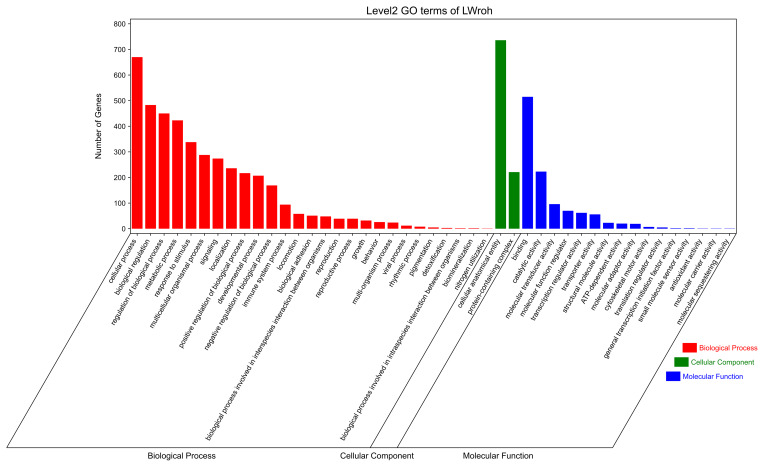
GO terms analysis of the candidate genes located in the Danish LW pig ROH islands. GO=gene ontology; LW=Large White; ROH=runs of homozygosity.

**Figure 4:**
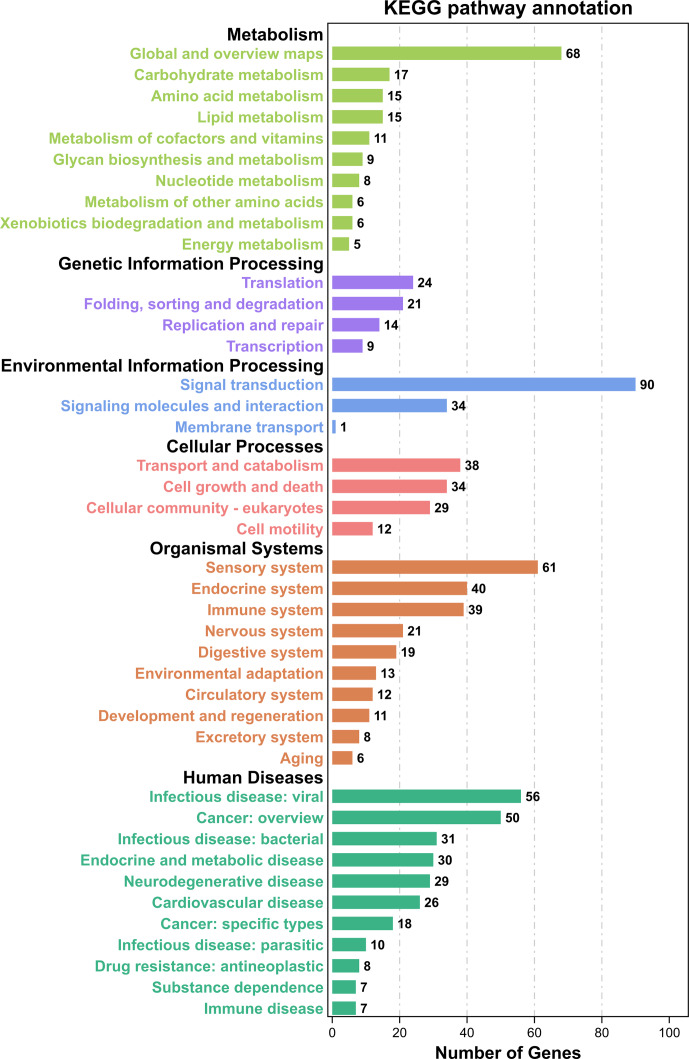
KEGG pathway annotation of candidate genes located in Danish LW pig ROH islands. KEGG=Kyoto Encyclopedia of Genes and Genomes; LW=Large White; ROH=runs of homozygosity.

GO and KEGG analyses were performed to evaluate the potential functions of candidate genes located in the ROH islands. A total of 1347 genes were enriched in 7530 GO terms, which were classified as biological processes (BPs, n=5624 ), cellular components (CCs, n=725 ), and molecular functions (MFs, n=1181 ). These candidate genes were primarily involved in cellular anatomical entities, cellular processes, binding, biological regulation, regulation of biological processes, and metabolic processes (Fig. 3). Subsequently, KEGG analysis showed that the candidate genes were enriched in 314 pathways, mainly involved in global and overview maps, translation, signal transduction, transport and catabolism, sensory systems, infectious diseases, and viruses (Fig. 4). We identified several pathways related to economic traits. The PI3K-Akt (ko04151), PPAR (ko03320), and fat digestion and absorption pathways (ko04975) are associated with growth and fat deposition. Prolactin signaling (ko04917), oocyte meiosis (ko04114), and progesterone-mediated oocyte maturation pathways (ko04914) are associated with reproductive performance. The Toll-like receptor (ko04620), Jak-STAT (ko04630), and MAPK signaling pathways (ko04010) are associated with immunity and disease resistance. However, none of these GO terms or KEGG pathways remained statistically significant.

**Figure 5:**
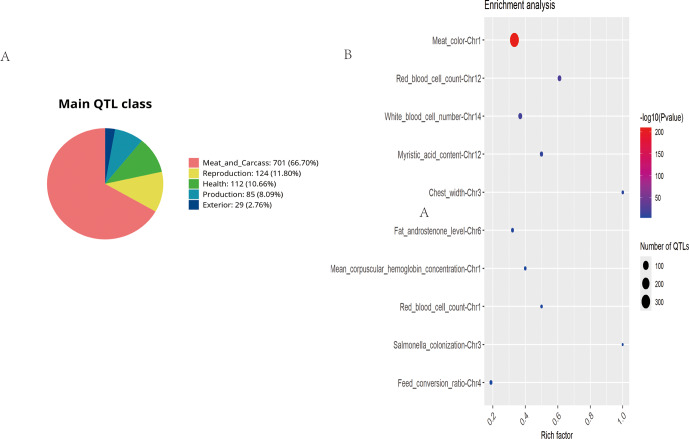
Summary of trait-associated QTLs mapped to ROH islands in Danish LW pigs. **(A)** Trait-based classification of annotated QTLs. **(B)** Significantly enriched QTLs displayed in a bubble plot format (false discovery rate-adjusted p value < 0.05). QTL=quantitative trait locus; ROH=runs of homozygosity; LW=Large White.

GO and KEGG enrichment analyses were considered limitations. Consequently, the pig QTL database was utilized for the functional annotation of ROH islands in Danish LW pigs (Fig. 5). A total of 1051 pig QTLs overlapped in the Danish LW pig ROH islands, among which the majority were associated with meat and carcass traits ( n=701 ), whereas the proportion of QTLs associated with external traits was the lowest ( n=29 ). The most enriched QTLs found per chromosome are shown in Fig. 5b. Specifically, the ROH islands in Danish LW pig chromosome 1 overlapped with QTLs associated with meat color, mean corpuscular hemoglobin concentration, and red blood cell count. The ROH island in Danish LW pig chromosome 3 overlapped with the QTLs associated with chest width and *Salmonella* colonization. On chromosome 4, the ROH island overlapped with QTLs associated with the feed conversion ratio. Additionally, some QTLs associated with blood indicators overlapped in the ROH islands on chromosomes 12 and 14.

We identified candidate genes that overlapped between the ROH islands in the present study and the selective sweep regions in the prior study (Wu et al., 2025). Subsequently, we conducted a literature review on the functions of these genes and identified reproductive performance-related genes (*ALDH1A2*, *APQ9*, *ACTG1*, *CDK6*, *ADAMTS9*, *PAPPA2*, and *ESR2*) and growth- and development-related genes (*NDN*, *CEP128*, *NFATC1*, *JAK2*, *KCNQ1*, *ANKRD22*, *ACTA2*, *FABP4*, *FAS*, *GDF15*, and *FGF21*).

## 4 Discussion

The pork industry holds a pivotal position in the Chinese agricultural sector and has a significant impact on the country's economy, food security, and cultural traditions. In 2023, the number of pigs slaughtered in China reached 726.624 million. Pork production reached 57.943 million tonnes, accounting for 59.4 % of total meat production (National Bureau of Statistics of China, https://www.stats.gov.cn, last access: 6 May 2025). Since the 1980s, lean-type pigs, represented by LW pigs, have become the dominant commercial breed in Chinese pig farms (Ai et al., 2013). Thus, a precise evaluation of the inbreeding level in LW pig populations and the identification of candidate genes related to their germplasm characteristics are crucial for future pig breeding projects. According to FAO24 guidelines, reliable genetic characterization requires a minimum of 15 samples for high-density markers and 20 samples for medium-density markers (Ajmone-Marsan et al., 2023). Although our sample size of 43 individuals was sufficient for initial ROH profiling, it may not have captured the full genetic diversity of the Danish LW population. Future studies with larger sample sizes will be necessary to validate these findings and improve the robustness of candidate gene identification.

In contrast to local pig breeds that have undergone prolonged natural selection, commercial pig breeds have a shorter history of domestication and have been subjected to strong artificial selection to achieve target phenotypic traits (Zhu et al., 2007; Wang et al., 2024). The effect of selection on the frequency of alleles associated with the selected genes results in the homozygosity of the corresponding alleles, and may lead to the formation of continuous homozygous fragments (Wientjes et al., 2024). It has been reported that ROH are not randomly generated and that population history and selection affect their distribution (Hewett et al., 2023). In the present study, we determined the distribution of ROH in the Danish LW pig population based on whole-genome resequencing data from 43 individuals, and the reliability of the results was ensured by high marker density. ROH fragments were abundant in the LW genome, and a total of 9446 ROH were identified, which is consistent with several previous reports on pigs and chickens, showing that commercial breeds have more ROH in their genomes (Jiang et al., 2022; Rostamzadeh Mahdabi et al., 2025). ROH information provides insights into population history and how genomic inbreeding affects chromosome architecture (Liu et al., 2024b). ROH were observed in all LW autosomes, but the number and length of ROH varied between chromosomes, with a positive trend observed for longer chromosomes with more ROH fragments. LW ROH were classified by setting different length ranges, and most ROH were short fragments, suggesting the occurrence of inbreeding in distant generations. However, the proportion of ROH ( >5Mb ) in LW pigs reached 10 %, higher than that of Baoshan, Ding'an, and other local pig breeds. This indicates strong selection pressure or inbreeding of LW pigs in recent generations (Li et al., 2024; Wang et al., 2025). Numerous studies have demonstrated that $F_{\text{ROH}}$ can more accurately reflect the true level of inbreeding than the breeding coefficient based on pedigree information, $F_{\text{PED}}$ (Shi et al., 2020; Schiavo et al., 2021). The average $F_{\text{ROH}}$ value of the Danish LW pig population was 0.24, which is less than that of Swiss LW pigs (Nosková et al., 2021). However, some Danish LW pigs have high $F_{\text{ROH}}$ values; therefore, these individuals should be excluded from further breeding programs to prevent inbreeding recession.

ROH islands are genomic regions with elevated homozygosity shared across individuals in a population, providing critical insights into the genetic architecture of artificial livestock selection and inbreeding. A previous study has shown that genome-specific loci and neighboring site homozygosity frequency increase with selection pressure, and ROH islands are strongly associated with selective sweeps driven by human-mediated breeding objectives (Macharia et al., 2024). ROH islands harbor candidate genes involved in important biological processes associated with growth rate, meat quality, and reproductive performance (Saleh et al., 2025; Sievers and Distl, 2025). In Tunchang pigs, ROH islands are enriched in genes related to reproduction (*HIRA*, *SERPIND1*), meat quality (*TBX1*, *PI4KA*), immunity (*RANBP1*, *ESS2*), and adaptation to heat stress (*DGCR8*, *TXNRD2*), reflecting historical selection for prolificacy and adaptation (Liu et al., 2024b). Similarly, Mangalitsa pigs have ROH islands containing genes linked to meat quality traits (*ABCA12*, *VIL1*, *PLSCR5*, *USP37*; Addo and Jung, 2022). In the present study, we investigated ROH islands in the Danish LW pig population and identified and annotated 1347 candidate genes. Functional enrichment analyses of these genes revealed no significant enrichment in GO terms or KEGG pathways. This may be because the ROH islands reflect selection for economically important traits that have been influenced by a large number of small-effect loci and the current limitations in porcine genome annotation. QTL are genomic regions associated with variations in complex traits. A large number of QTL sites were found in the Danish LW pig ROH islands, most of which were related to growth (longissimus muscle depth, average daily gain, body weight, and feed conversion ratio) and reproductive performance (offspring number, litter weight, piglets born alive, and teat number), which is consistent with the breeding direction of LW pigs.

In previous studies, we identified the genome-wide selection signals of LW pigs and found 20 genes in both selected regions and the ROH islands of the pigs. In particular, *ALDH1A2* and* APQ9* were associated with pig litter size and pregnancy; *NDN*, *CEP128*, and *NFATC1* were associated with pig growth and development; and the correlation between selection pressure and ROH island formation was further emphasized (Wu et al., 2025). Then, based on GO and KEGG functional analysis and QTL overlapping results, and following a comprehensive literature search, we identified several other candidate genes that are associated with reproductive performance in the LW pig ROH islands. *ACTG1*, *CDK6*, and *ADAMTS9* genes have been reported and play important roles in mammalian ovarian remodeling and follicle development (Ataei-Nazari et al., 2022; Zhang et al., 2022; Li et al., 2023). The *PAPPA2* gene was identified as being linked to litter traits in Yorkshire and Landrace pigs in a genome-wide association study (Zhao et al., 2022). Polymorphisms in the *ESR2* gene are associated with the weaning-to-estrus interval and the number of piglets born dead (Rempel et al., 2010).

We screened several genes related to the growth and development of LW pigs. As a paternally expressed imprinted gene, *KCNQ1* is associated with pathways related to glycogen metabolism, digestion, and protein absorption, which are key candidate genes for feed conversion in commercial pigs (Ma et al., 2024; Xiang et al., 2024). The *JAK2* gene plays a key role in the growth axis signaling pathway and is associated with animal growth and development. Individual cattle and sheep with specific genotypes of *JAK2* polymorphic loci had the highest body weights and daily gains during the study period, and* JAK2* was differentially expressed in the hypothalamus of high- and low-feed-efficiency LW pigs (Hou et al., 2018; Oster et al., 2023). A 100 kg live weight (AGE100) reflected the growth rate of the pig, and Zhou et al. (2023) reported that *ANKRD22* partially overlaps a potentially significant SNP (SSC14_101189804) related to AGE100 in LW pigs. *ACTA2*, *FABP4*, and *FAS* genes play critical roles in adipocyte differentiation and lipid metabolism, and may be candidate core genes related to intramuscular fat deposition (Sun et al., 2024; Yang et al., 2024; Yi et al., 2024). *GDF15* and *FGF21* genes are associated with lipid metabolism and liver function, and affect fat deposits and body weight (Xu et al., 2025).

Although our sample size of 43 individuals was sufficient for initial ROH profiling, it may not have captured the full genetic diversity of the Danish LW population. Future studies with larger cohorts are recommended to validate these findings and improve the robustness of candidate gene identification.

## 5 Conclusions

In summary, we used whole-genome resequencing data to characterize the distribution and occurrence of ROH in Danish LW pigs and obtained the numbers, length, $F_{\text{ROH}}$ , and high-frequency region. Our results revealed that artificial selection pressures have a certain influence on ROH in commercial breeds, resulting in an increased proportion of long ROH fragments. Furthermore, we identified several candidate genes associated with reproductive and growth performance in ROH islands. Our findings will help to elucidate the potential molecular marker locus and selection history of Danish LW pigs and will provide an important reference for future breeding and genetic improvement plans.

## Supplement

10.5194/aab-69-25-2026-supplement The supplement related to this article is available online at https://doi.org/10.5194/aab-69-25-2026-supplement.

## Data Availability

Data sets generated during the current study are available from the corresponding author on reasonable request.
