# Supplementary material for: Runs of homozygosity reveal candidate genes for economic traits in Danish Large White pigs
Source: Arch Anim Breed. 2026 Jan 12;69(1):25–35. doi: 10.5194/aab-69-25-2026 (PMC13100821; doi:10.5194/aab-69-25-2026)
Supplement: The supplement related to this article is available online at https://doi.org/10.5194/aab-69-25-2026-supplement. [file aab-69-25-2026-supplement.pdf]

Supplement of Arch. Anim. Breed., 69, 25–35, 2026
https://doi.org/10.5194/aab-69-25-2026-supplement
© Author(s) 2026. CC BY 4.0 License.

Open Access
Archives Animal Breeding

Supplement of

Runs of homozygosity reveal candidate genes for economic traits in Danish Large White pigs

Weimin Ding et al.

Correspondence to: Zongjun Yin (yinzongjun@ahau.edu.cn)

The copyright of individual parts of the supplement might differ from the article licence.

Table S1. Genomic kinship matrix of the 43 Danish Large White pigs					
IID1	IID2	NSNP	HETHET	IBS0	KINSHIP
LW2YEHA	LW2YEBA	10441702	0.151673	0.0601952	0.0476055
LW3YVCA	LW3YVBA	10255874	0.102719	0.0586651	-0.031905
LW0YGCA	LW0YGAA	10443072	0.0956214	0.064792	-0.071493
LW3YKGA	LW2YKCA	10410003	0.100416	0.0884178	-0.138645
LW3YXDA	LW3YXBA	10153871	0.0856791	0.0650876	-0.163625
LW3YVBA	LW1YI2A	10424278	0.0788108	0.0730242	-0.164442
LW2YKCA	LW2YD1A	10421164	0.10315	0.0973369	-0.171528
LW1YYAA	LW0YIBA	10442844	0.0953617	0.0961534	-0.174767
LW3YVBA	LW1YR2A	10429227	0.0813382	0.0829829	-0.184445
LW3YRDA	LW2YD1A	10463529	0.0958828	0.097468	-0.185501
LW3YA1A	LW2YA1A	10471866	0.0714889	0.0772717	-0.192426
LW0YVKA	LW0YCBA	10390856	0.0855908	0.0885889	-0.197911
LW2YKCA	LW2YEBA	10396615	0.099421	0.105544	-0.207929
LW3YJBA	LW1YQBA	10430579	0.0829616	0.0805906	-0.208023
LW2YA1A	LW0YBFA	10445031	0.0784543	0.0845171	-0.208629
LW3YRDA	LW2YKCA	10415576	0.0922565	0.101001	-0.213063
LW3YRDA	LW1YS2A	10480019	0.0883339	0.101651	-0.213646
LW3YKGA	LW2YEBA	10432448	0.0962066	0.104987	-0.219414
LW3YRDA	LW0YCBA	10380548	0.085808	0.101113	-0.220149
LW2YKCA	LW2YEHA	10415795	0.101398	0.108934	-0.22026
LW1YS2A	LW1YA2A	10505564	0.0894666	0.0914687	-0.223863
LW3YXDA	LW2YR2A	10488096	0.0738496	0.0855318	-0.230996
LW0YVKA	LW0YPDA	10409778	0.0789903	0.0963314	-0.231381
LW0YXBA	LW0YHCA	10485975	0.0788713	0.0846601	-0.233577
LW3YKGA	LW2YD1A	10457574	0.0919358	0.111019	-0.235878
LW3YRDA	LW3YKGA	10452846	0.0907676	0.107717	-0.235942
LW1YS2A	LW1YQBA	10473220	0.0906275	0.0995923	-0.237448
LW1YS2A	LW0YIBA	10488063	0.0936859	0.109902	-0.237911
LW2YEBA	LW1YYAA	10415849	0.098	0.108114	-0.238554
LW3YMGA	LW2YEHA	10456674	0.099328	0.108998	-0.23928
LW3YKGA	LW2YEHA	10452588	0.0957534	0.109837	-0.241189
LW2YEHA	LW1YYAA	10434863	0.100798	0.109824	-0.243795
LW0YNAA	LW0YIBA	10350017	0.090332	0.10194	-0.253253
LW0YPDA	LW0YCBA	10324246	0.0827274	0.0995679	-0.253658
LW3YMGA	LW2YEBA	10436220	0.0956868	0.11353	-0.258052
LW2YKCA	LW1YS2A	10436909	0.0852951	0.108865	-0.259191
LW0YIBA	LW0YCBA	10388374	0.0885426	0.112359	-0.260161
LW0YXBA	LW0YNAA	10347914	0.0769707	0.10263	-0.261844
LW2YKCA	LW0YIBA	10423222	0.0923137	0.116516	-0.262405
LW0YRBA	LW0YPDA	10278749	0.0787766	0.0996233	-0.263417
LW3YVCA	LW0YWCA	10272764	0.0746939	0.0870487	-0.26413
LW2YEHA	LW2YD1A	10463772	0.0977726	0.115268	-0.264436

LW3YXDA	LW0YNAA	10355131	0.0799963	0.10049	-0.266419
LW3YKGA	LW1YYAA	10428301	0.0906528	0.117819	-0.266908
LW2YA1A	LW0YKFA	10448813	0.0789598	0.0991961	-0.26829
LW3YMGA	LW1YTFA	10457191	0.0851325	0.107619	-0.26876
LW1YR2A	LW1YI2A	10500957	0.0719853	0.0964923	-0.271244
LW3YSDA	LW3YMGA	10115705	0.085881	0.100786	-0.271269
LW3YMGA	LW2YKCA	10413031	0.09115	0.119315	-0.271448
LW3YMGA	LW1YYAA	10432371	0.0910077	0.120533	-0.272019
LW3YHAA	LW2YKCA	10407133	0.0910878	0.112457	-0.274285
LW3YRDA	LW0YIBA	10465708	0.0811229	0.115402	-0.275707
LW3YVCA	LW1YR2A	10329144	0.0728779	0.1015	-0.275869
LW3YMGA	LW0YIBA	10463819	0.0906204	0.12192	-0.276691
LW3YRDA	LW1YA2A	10482263	0.0829835	0.100318	-0.276734
LW2YKCA	LW0YCBA	10338850	0.0889492	0.114423	-0.277101
LW1YA2A	LW0YXBA	10488323	0.0772342	0.102085	-0.277911
LW1YS2A	LW0YGAA	10473287	0.0862627	0.102791	-0.279885
LW3YHAA	LW0YCBA	10372972	0.0807603	0.113718	-0.280172
LW3YVCA	LW1YI2A	10323471	0.0743872	0.0981948	-0.28024
LW2YEBA	LW2YD1A	10443274	0.0970053	0.120411	-0.280347
LW1YR2A	LW0YGCA	10472863	0.0760651	0.105191	-0.280584
LW2YD1A	LW1YS2A	10484896	0.0856375	0.117224	-0.280817
LW1YS2A	LW0YCBA	10401974	0.0809697	0.115835	-0.280838
LW1YQBA	LW0YKFA	10460856	0.0783542	0.108462	-0.28174
LW3YMGA	LW3YKGA	10449627	0.0903188	0.123227	-0.282376
LW1YR2A	LW0YXBA	10488013	0.0762682	0.10228	-0.282739
LW3YMGA	LW3YHAA	10446926	0.0883856	0.115746	-0.283315
LW3YMGA	LW2YD1A	10460846	0.0885378	0.124529	-0.286245
LW3YA1A	LW0YBFA	10496777	0.0696784	0.100297	-0.286373
LW3YSDA	LW1YTFA	10118711	0.0770439	0.104979	-0.286546
LW1YR2A	LW0YGAA	10475400	0.0731708	0.105937	-0.288294
LW1YS2A	LW0YGCA	10470672	0.0788159	0.101031	-0.289557
LW3YHAA	LW0YXBA	10455029	0.0809179	0.112833	-0.289696
LW1YS2A	LW0YKFA	10490119	0.0825774	0.112371	-0.292022
LW0YXBA	LW0YCBA	10386015	0.0758663	0.110328	-0.293239
LW0YKFA	LW0YCBA	10390783	0.0796591	0.111832	-0.293405
LW2YEHA	LW0YIBA	10466007	0.0927698	0.120343	-0.294627
LW2YEBA	LW0YIBA	10446103	0.0950343	0.123246	-0.297362
LW1YS2A	LW1YR2A	10503749	0.0789989	0.103524	-0.297382
LW0YGAA	LW0YCBA	10374055	0.0763116	0.102932	-0.298783
LW1YI2A	LW0YWCA	10442488	0.0671193	0.0946563	-0.298955
LW3YHAA	LW2YEBA	10430811	0.0889446	0.113794	-0.299402
LW3YHAA	LW0YIBA	10456865	0.0854868	0.119102	-0.299503
LW2YEBA	LW0YCBA	10362543	0.0905823	0.117039	-0.299643
LW1YA2A	LW0YBFA	10488994	0.0749692	0.102938	-0.300292
LW1YYAA	LW0YCBA	10357422	0.0856425	0.121914	-0.300337
LW1YS2A	LW0YHCA	10500860	0.0863034	0.099918	-0.300581

LW3YHAA	LW1YS2A	10470948	0.0826769	0.119614	-0.300728
LW0YRBA	LW0YCBA	10259817	0.0786539	0.103514	-0.300811
LW1YS2A	LW0YNAA	10363224	0.0814162	0.111148	-0.301055
LW3YRDA	LW0YDCA	10463811	0.0763191	0.0999846	-0.301127
LW0YXBA	LW0YIBA	10471304	0.0799091	0.112364	-0.301649
LW0YXBA	LW0YGCA	10454269	0.0760773	0.106265	-0.301692
LW2YD1A	LW0YCBA	10385135	0.0851256	0.121617	-0.302305
LW2YD1A	LW1YTFA	10464309	0.0819523	0.114764	-0.302333
LW3YVCA	LW1YS2A	10326254	0.0781997	0.107665	-0.302602
LW3YMGA	LW0YCBA	10377989	0.0876226	0.12392	-0.306254
LW3YHAA	LW1YYAA	10425552	0.0860979	0.12151	-0.307293
LW3YMGA	LW0YNAA	10339697	0.0837853	0.112176	-0.308519
LW1YS2A	LW0YXBA	10485409	0.0840829	0.117964	-0.309286
LW0YGCA	LW0YCBA	10371007	0.0791984	0.106563	-0.309693
LW3YKGA	LW0YCBA	10374225	0.0846983	0.12236	-0.309931
LW3YVBA	LW0YGCA	10395367	0.0720297	0.108233	-0.310143
LW3YRDA	LW2YEHA	10457924	0.0927814	0.12261	-0.310462
LW2YKCA	LW1YYAA	10391698	0.0906383	0.128834	-0.310649
LW1YQBA	LW0YCBA	10372443	0.0798389	0.113116	-0.31076
LW2YEHA	LW0YCBA	10380602	0.0934818	0.120436	-0.311012
LW2YD1A	LW0YIBA	10470661	0.0852359	0.129156	-0.312058
LW1YFBA	LW0YXBA	10482426	0.0719938	0.107398	-0.312121
LW2YIGA	LW1YS2A	10506252	0.0800271	0.0980007	-0.312182
LW3YXDA	LW1YS2A	10493095	0.0822955	0.107722	-0.312754
LW3YMGA	LW1YS2A	10477043	0.0864261	0.126862	-0.314766
LW0YRBA	LW0YKFA	10345472	0.0723233	0.107169	-0.315356
LW2YD1A	LW1YYAA	10438658	0.0834291	0.129017	-0.315863
LW1YR2A	LW0YWCA	10447895	0.0730302	0.0989767	-0.316105
LW3YHAA	LW1YTFA	10449881	0.0817214	0.121845	-0.316264
LW3YXDA	LW0YGCA	10461822	0.0708844	0.110932	-0.316866
LW3YVBA	LW0YCBA	10327137	0.0793272	0.113766	-0.317347
LW1YA2A	LW0YGCA	10472910	0.0723967	0.111967	-0.317871
LW1YA2A	LW0YCBA	10406434	0.0777293	0.10912	-0.31872
LW3YRDA	LW0YGAA	10450690	0.0827164	0.109719	-0.31876
LW3YPCA	LW2YIGA	10479855	0.0720046	0.105927	-0.319375
LW2YQ1A	LW0YKFA	10498953	0.0799613	0.10406	-0.31949
LW3YSDA	LW3YHAA	10110682	0.0796532	0.113063	-0.319493
LW3YVCA	LW0YGCA	10295634	0.0719276	0.111167	-0.320094
LW2YIGA	LW2YA1A	10463917	0.0655652	0.101065	-0.320784
LW2YEBA	LW0YVKA	10448109	0.0865116	0.111119	-0.32087
LW0YVKA	LW0YRBA	10345113	0.0752811	0.110818	-0.320871
LW3YRDA	LW0YXBA	10463271	0.0846714	0.120615	-0.321117
LW3YHAA	LW2YEHA	10449702	0.0914591	0.120085	-0.322601
LW3YA1A	LW0YNAA	10375329	0.0723932	0.10456	-0.322746
LW3YHAA	LW0YNAA	10334833	0.0736117	0.114266	-0.3229
LW2YD1A	LW0YNAA	10346647	0.0798345	0.113717	-0.322913

LW3YVBA	LW1YQBA	10396932	0.0719706	0.116228	-0.32295
LW0YNAA	LW0YCBA	10268441	0.0800704	0.116575	-0.323584
LW3YHAA	LW2YD1A	10453854	0.0861984	0.125543	-0.323938
LW1YTFA	LW0YCBA	10381490	0.0788467	0.121789	-0.324787
LW2YIGA	LW0YBFA	10489847	0.0579955	0.100646	-0.32491
LW3YVBA	LW0YNAA	10288907	0.0729795	0.117271	-0.32527
LW1YA2A	LW0YNAA	10368905	0.0731111	0.113014	-0.325466
LW3YXDA	LW1YA2A	10495849	0.0720921	0.113132	-0.325493
LW3YVBA	LW1YS2A	10425518	0.0756004	0.113465	-0.325736
LW3YVBA	LW0YXBA	10409543	0.0721094	0.115435	-0.325744
LW3YVCA	LW0YGAA	10298717	0.0694254	0.111867	-0.326281
LW3YVCA	LW3YPCA	10301348	0.0695528	0.105894	-0.326756
LW2YA1A	LW1YQBA	10430785	0.076352	0.113247	-0.326811
LW3YPCA	LW0YNAA	10337636	0.0717655	0.106024	-0.327013
LW3YRDA	LW1YYAA	10433322	0.0845534	0.131376	-0.327386
LW3YRDA	LW3YMGA	10455109	0.0810163	0.129502	-0.328612
LW3YKGA	LW0YIBA	10459297	0.0874064	0.133862	-0.329073
LW3YVCA	LW1YQBA	10297224	0.0747745	0.117836	-0.329702
LW2YA1A	LW1YS2A	10459573	0.0831969	0.112083	-0.329888
LW0YXBA	LW0YGAA	10456530	0.0780694	0.114416	-0.329928
LW3YPCA	LW1YS2A	10475900	0.0802168	0.105781	-0.330011
LW3YVCA	LW0YNAA	10191899	0.0704391	0.115943	-0.330383
LW3YRDA	LW2YEBA	10437259	0.0893823	0.127979	-0.33222
LW1YS2A	LW0YBFA	10485764	0.0800872	0.105522	-0.33276
LW1YFBA	LW0YGCA	10466957	0.0644803	0.111976	-0.332769
LW2YKCA	LW1YTFA	10416265	0.0821356	0.120901	-0.333459
LW1YS2A	LW0YDCA	10485369	0.0777605	0.10937	-0.334841
LW3YQAA	LW1YQBA	10230024	0.0714183	0.0966764	-0.334875
LW3YXDA	LW3YPCA	10466877	0.0676743	0.109195	-0.336215
LW1YQBA	LW0YXBA	10456312	0.0819465	0.123728	-0.336306
LW3YJBA	LW2YIGA	10462140	0.0601215	0.104489	-0.336565
LW1YR2A	LW1YFBA	10500203	0.0666821	0.114618	-0.336706
LW1YTFA	LW0YXBA	10464442	0.0771628	0.124701	-0.337269
LW1YQBA	LW0YGCA	10441484	0.0712111	0.114104	-0.337542
LW1YTFA	LW0YIBA	10466874	0.0826678	0.12489	-0.337936
LW2YR2A	LW0YNAA	10357161	0.0715253	0.105485	-0.337968
LW3YHAA	LW0YVKA	10458953	0.0742332	0.119532	-0.338311
LW2YA1A	LW0YGCA	10428725	0.0729785	0.116993	-0.338419
LW1YA2A	LW0YDCA	10487871	0.0687718	0.113378	-0.339011
LW1YYAA	LW1YS2A	10455427	0.0801507	0.130893	-0.33921
LW1YA2A	LW0YGAA	10476007	0.0726764	0.117706	-0.339237
LW2YKCA	LW0YXBA	10420709	0.0815436	0.12019	-0.339269
LW1YA2A	LW0YIBA	10490660	0.0799916	0.113117	-0.339296
LW3YKGA	LW3YHAA	10443189	0.0839844	0.127418	-0.339323
LW0YDCA	LW0YBFA	10469118	0.0679026	0.109745	-0.339372
LW2YQ1A	LW2YIGA	10514016	0.0652221	0.108408	-0.339563

LW2YKCA	LW0YNAA	10300680	0.0822467	0.116927	-0.339762
LW3YPCA	LW0YBFA	10459266	0.0686037	0.111886	-0.340365
LW1YTFA	LW1YS2A	10480633	0.0845135	0.128258	-0.341153
LW0YPDA	LW0YNAA	10285907	0.0751875	0.122389	-0.341414
LW3YSDA	LW1YA2A	10142762	0.0706953	0.117276	-0.341614
LW1YR2A	LW0YNAA	10365725	0.0699587	0.114867	-0.341667
LW3YPCA	LW1YQBA	10446632	0.0762384	0.111801	-0.342008
LW3YVBA	LW0YGAA	10397031	0.0732579	0.116939	-0.342047
LW0YGAA	LW0YBFA	10456814	0.0662622	0.10865	-0.342807
LW2YA1A	LW1YA2A	10462214	0.0706612	0.116446	-0.34326
LW1YI2A	LW0YGCA	10467823	0.0718111	0.113326	-0.343491
LW3YXBA	LW2YR2A	10153806	0.0648608	0.0935935	-0.34401
LW3YRDA	LW1YTFA	10458309	0.0755571	0.123969	-0.344144
LW2YKCA	LW0YKFA	10425303	0.082349	0.121461	-0.344292
LW0YXBA	LW0YKFA	10473188	0.0786591	0.127684	-0.34498
LW2YD1A	LW0YKFA	10473388	0.0820795	0.123813	-0.345051
LW3YXDA	LW3YVCA	10318229	0.069549	0.11572	-0.346446
LW1YR2A	LW0YCBA	10404168	0.0759726	0.114549	-0.346819
LW0YNAA	LW0YKFA	10352000	0.0709663	0.121194	-0.346846
LW2YR2A	LW1YS2A	10495632	0.0706542	0.102098	-0.347583
LW3YXDA	LW3YA1A	10503907	0.0682313	0.111317	-0.348291
LW3YJBA	LW1YI2A	10455300	0.064899	0.107606	-0.3485
LW3YXDA	LW2YA1A	10451116	0.0718225	0.119171	-0.348766
LW2YD1A	LW1YA2A	10487228	0.0763349	0.113248	-0.349075
LW0YCBA	LW0YBFA	10386606	0.0726568	0.106128	-0.349108
LW3YMGA	LW0YKFA	10465682	0.0839868	0.125754	-0.349321
LW3YMGA	LW0YXBA	10461368	0.0821727	0.12541	-0.349519
LW1YQBA	LW1YA2A	10474130	0.0719526	0.118476	-0.349688
LW1YYAA	LW1YTFA	10435009	0.0828126	0.128192	-0.349694
LW2YIGA	LW0YGCA	10474464	0.0652523	0.107132	-0.350496
LW3YPCA	LW2YR2A	10470145	0.0687817	0.112118	-0.35062
LW3YA1A	LW1YS2A	10512718	0.0744594	0.106786	-0.350655
LW2YEHA	LW1YS2A	10479375	0.0847231	0.128107	-0.350748
LW3YVBA	LW2YKCA	10361575	0.0828553	0.119088	-0.350798
LW3YVCA	LW0YCBA	10229470	0.0817147	0.121872	-0.351048
LW0YKFA	LW0YGCA	10458867	0.0763143	0.118453	-0.351179
LW3YPCA	LW3YA1A	10486718	0.0659477	0.112977	-0.351264
LW2YR2A	LW0YBFA	10479068	0.0659368	0.111436	-0.352176
LW3YHAA	LW0YKFA	10459260	0.0754761	0.12582	-0.352294
LW1YS2A	LW1YFBA	10497733	0.0729196	0.114008	-0.352903
LW3YXDA	LW0YBFA	10476635	0.0693763	0.112981	-0.353133
LW2YR2A	LW0YWCA	10439139	0.0651296	0.107024	-0.353335
LW3YRDA	LW1YQBA	10451042	0.080406	0.123038	-0.353422
LW2YD1A	LW1YQBA	10455736	0.0789016	0.121004	-0.353706
LW3YVCA	LW0YIBA	10313137	0.0785125	0.118861	-0.353729
LW2YEHA	LW0YXBA	10463573	0.0872976	0.121332	-0.353913

LW2YR2A	LW2YIGA	10499347	0.060237	0.10945	-0.355539
LW2YD1A	LW0YXBA	10468374	0.0789815	0.125472	-0.355839
LW3YSDA	LW0YNAA	10010273	0.0717965	0.121746	-0.356074
LW1YQBA	LW0YIBA	10458314	0.0819268	0.123594	-0.356543
LW2YR2A	LW0YGCA	10464988	0.0675387	0.110445	-0.356634
LW3YSDA	LW2YKCA	10077267	0.0820996	0.117561	-0.356761
LW2YEHA	LW1YTFA	10458802	0.0866969	0.123746	-0.357014
LW3YVBA	LW0YIBA	10412017	0.0792133	0.12153	-0.357094
LW3YPCA	LW0YWCA	10419495	0.066646	0.107457	-0.357506
LW3YRDA	LW0YPDA	10398898	0.0804046	0.123254	-0.357695
LW1YFBA	LW0YGAA	10468873	0.066173	0.11933	-0.358082
LW0YGCA	LW0YBFA	10453960	0.068852	0.113667	-0.358291
LW1YQBA	LW0YNAA	10332823	0.072405	0.12609	-0.35904
LW1YR2A	LW1YQBA	10474399	0.0774291	0.122964	-0.359391
LW2YR2A	LW1YA2A	10498105	0.0651332	0.109457	-0.359482
LW2YEBA	LW1YTFA	10439228	0.0871874	0.126	-0.360072
LW3YRDA	LW1YR2A	10481922	0.0790807	0.118088	-0.360148
LW3YHAA	LW1YA2A	10474722	0.0760247	0.119279	-0.360227
LW0YRBA	LW0YBFA	10340907	0.0666396	0.113387	-0.36034
LW2YA1A	LW0YNAA	10321517	0.0698733	0.118023	-0.360493
LW3YHAA	LW0YPDA	10391147	0.0728221	0.122355	-0.360529
LW2YQ1A	LW0YBFA	10493846	0.0647963	0.112705	-0.360738
LW3YPCA	LW0YKFA	10464112	0.0675209	0.110422	-0.360771
LW3YVCA	LW0YXBA	10310015	0.0721999	0.12277	-0.36107
LW3YXDA	LW0YXBA	10476530	0.0738838	0.118869	-0.361422
LW2YA1A	LW0YXBA	10442982	0.0763315	0.11996	-0.361505
LW3YRDA	LW3YHAA	10448595	0.0753173	0.131562	-0.361768
LW3YRDA	LW1YFBA	10476105	0.0762957	0.117288	-0.362075
LW2YEHA	LW0YVKA	10468161	0.0887933	0.121654	-0.362472
LW1YFBA	LW1YA2A	10500797	0.0660374	0.120491	-0.362911
LW3YVBA	LW0YPDA	10345629	0.0701669	0.125682	-0.362912
LW1YR2A	LW1YA2A	10506249	0.0665074	0.12067	-0.363354
LW3YVCA	LW3YHAA	10296058	0.0785062	0.124394	-0.363744
LW3YHAA	LW1YQBA	10441514	0.0725048	0.123868	-0.363958
LW3YHAA	LW1YFBA	10467417	0.0775133	0.120575	-0.364431
LW3YVBA	LW2YA1A	10384349	0.0700574	0.119581	-0.364655
LW2YA1A	LW0YIBA	10445926	0.0791592	0.116908	-0.364759
LW3YXDA	LW0YGAA	10463777	0.0660836	0.119908	-0.364951
LW3YPCA	LW1YR2A	10477835	0.0712436	0.117161	-0.36553
LW1YR2A	LW0YBFA	10487413	0.0685592	0.114982	-0.365588
LW3YVCA	LW2YKCA	10263157	0.0825122	0.121108	-0.365599
LW1YA2A	LW0YHCA	10503885	0.0738551	0.115894	-0.365702
LW3YVBA	LW3YRDA	10403480	0.0724928	0.121325	-0.365797
LW3YVBA	LW0YWCA	10370346	0.0688323	0.105603	-0.365856
LW3YVCA	LW0YPDA	10247585	0.0663692	0.123164	-0.366399
LW2YR2A	LW1YR2A	10497705	0.0699589	0.113754	-0.366838

LW3YRDA	LW0YGCA	10448487	0.0750278	0.117097	-0.366964
LW1YFBA	LW0YCBA	10398415	0.0758324	0.119559	-0.367336
LW2YA1A	LW0YRBA	10315318	0.0660432	0.120956	-0.367713
LW0YKFA	LW0YBFA	10473898	0.0728906	0.113866	-0.36793
LW2YEBA	LW1YS2A	10459641	0.0876124	0.135251	-0.368086
LW3YPCA	LW1YI2A	10472538	0.0670536	0.118154	-0.36834
LW0YHCA	LW0YBFA	10484604	0.0686918	0.117246	-0.368398
LW1YQBA	LW0YBFA	10456065	0.0758001	0.116811	-0.368415
LW3YPCA	LW0YGCA	10444055	0.0685924	0.116869	-0.368719
LW2YKCA	LW0YPDA	10357372	0.0830872	0.123737	-0.369052
LW1YTFA	LW1YQBA	10451150	0.0740115	0.127369	-0.369059
LW1YTFA	LW1YR2A	10482645	0.0742568	0.12145	-0.369157
LW3YXDA	LW0YKFA	10481588	0.0725234	0.120337	-0.369627
LW3YKGA	LW1YS2A	10472977	0.0817774	0.138632	-0.370196
LW1YYAA	LW0YXBA	10439240	0.0792553	0.129833	-0.370286
LW0YWCA	LW0YBFA	10429512	0.0670237	0.110794	-0.370321
LW3YPCA	LW0YRBA	10331517	0.0663866	0.116508	-0.370343
LW3YXDA	LW0YDCA	10476334	0.0675572	0.120916	-0.37042
LW3YVCA	LW3YRDA	10304121	0.0725212	0.120926	-0.370484
LW0YNAA	LW0YGCA	10331778	0.0697581	0.12114	-0.370871
LW3YA1A	LW2YIGA	10517027	0.0665584	0.115206	-0.370893
LW2YQ1A	LW1YS2A	10509685	0.0741588	0.108977	-0.371076
LW2YA1A	LW0YCBA	10360564	0.0729139	0.117541	-0.371444
LW2YKCA	LW1YA2A	10439961	0.0789616	0.117742	-0.371649
LW0YDCA	LW0YCBA	10385727	0.0742015	0.116939	-0.371748
LW3YMGA	LW1YQBA	10447955	0.0818076	0.127006	-0.371985
LW2YQ1A	LW1YQBA	10480192	0.0713568	0.113061	-0.371995
LW1YYAA	LW0YVKA	10443667	0.0746566	0.125389	-0.372139
LW1YQBA	LW1YI2A	10470067	0.0715265	0.117504	-0.373243
LW1YTFA	LW0YNAA	10342789	0.0738492	0.128264	-0.373268
LW0YIBA	LW0YDCA	10471573	0.0794343	0.117705	-0.373318
LW3YHAA	LW2YA1A	10428852	0.070481	0.117803	-0.37343
LW3YKGA	LW1YTFA	10452419	0.0819101	0.132346	-0.37384
LW3YVCA	LW2YA1A	10285129	0.0678842	0.121413	-0.373904
LW0YXBA	LW0YBFA	10469064	0.073315	0.115415	-0.374814
LW1YFBA	LW0YHCA	10497523	0.0613788	0.112105	-0.374953
LW2YD1A	LW0YVKA	10472573	0.0831423	0.129773	-0.37506
LW2YKCA	LW0YVKA	10424453	0.0809766	0.126519	-0.375405
LW0YXBA	LW0YPDA	10404603	0.0737981	0.128805	-0.375721
LW3YKGA	LW1YFBA	10470332	0.0790264	0.119515	-0.37596
LW3YPCA	LW0YGAA	10446863	0.0718443	0.120001	-0.376151
LW2YEBA	LW0YNAA	10324559	0.0809924	0.121307	-0.376258
LW2YKCA	LW1YQBA	10407410	0.0810351	0.125491	-0.376453
LW3YRDA	LW0YNAA	10340603	0.0768007	0.127068	-0.376756
LW2YEHA	LW0YNAA	10342131	0.0820435	0.120828	-0.376992
LW1YQBA	LW0YGAA	10443183	0.0717705	0.124347	-0.376998

LW1YYAA	LW0YPDA	10376126	0.0778598	0.126003	-0.377023
LW2YIGA	LW1YI2A	10502529	0.0645027	0.114931	-0.377374
LW2YA1A	LW1YR2A	10461827	0.0723353	0.126168	-0.379105
LW3YJBA	LW2YQ1A	10465154	0.0669278	0.117079	-0.379456
LW0YIBA	LW0YGCA	10456343	0.0789364	0.121158	-0.379491
LW1YS2A	LW1YI2A	10498684	0.0788427	0.117381	-0.379937
LW3YXDA	LW0YCBA	10393250	0.072873	0.119691	-0.380016
LW3YVCA	LW0YRBA	10184984	0.0711738	0.124417	-0.380023
LW3YKGA	LW0YVKA	10462498	0.0798281	0.128394	-0.38039
LW3YHAA	LW1YR2A	10473118	0.0698997	0.120739	-0.381036
LW1YTFA	LW1YA2A	10483170	0.0737656	0.12462	-0.381179
LW2YA1A	LW0YDCA	10443438	0.0750144	0.127323	-0.381796
LW2YIGA	LW1YR2A	10507756	0.0618801	0.1123	-0.381896
LW3YVBA	LW1YTFA	10405054	0.0753535	0.130365	-0.382143
LW3YXDA	LW3YHAA	10462326	0.071892	0.12075	-0.382169
LW2YEBA	LW0YPDA	10380826	0.0835631	0.122998	-0.382175
LW3YSDA	LW1YYAA	10095421	0.0794586	0.125358	-0.38227
LW3YVBA	LW3YPCA	10399851	0.0663361	0.116576	-0.382338
LW1YI2A	LW0YCBA	10398223	0.078249	0.117997	-0.38244
LW3YA1A	LW1YA2A	10515622	0.0692778	0.119005	-0.382474
LW0YNAA	LW0YBFA	10347945	0.0690601	0.11664	-0.382477
LW3YVBA	LW3YMGA	10401888	0.0808077	0.128218	-0.3825
LW3YPCA	LW0YXBA	10459098	0.0715645	0.117299	-0.382594
LW3YXDA	LW1YR2A	10495120	0.0716169	0.126781	-0.382614
LW3YPCA	LW2YQ1A	10483174	0.0590008	0.114533	-0.382801
LW3YRDA	LW0YKFA	10467566	0.076396	0.132066	-0.382984
LW2YQ1A	LW0YRBA	10364598	0.0677443	0.116705	-0.383187
LW3YJBA	LW1YS2A	10457503	0.073053	0.109557	-0.383746
LW3YHAA	LW0YBFA	10455031	0.0688034	0.113109	-0.383845
LW3YVCA	LW0YBFA	10311012	0.0701909	0.118708	-0.384525
LW1YFBA	LW0YNAA	10359310	0.0685538	0.124896	-0.385082
LW3YVBA	LW3YHAA	10395360	0.0732999	0.128704	-0.385151
LW1YI2A	LW0YGAA	10468858	0.0696373	0.121663	-0.385175
LW0YNAA	LW0YGAA	10335476	0.0698321	0.125274	-0.385353
LW2YKCA	LW2YA1A	10394818	0.0761588	0.117799	-0.385692
LW3YMGA	LW2YA1A	10435534	0.0759674	0.119996	-0.38579
LW3YXDA	LW3YVBA	10417370	0.070751	0.125154	-0.386009
LW2YR2A	LW2YA1A	10453405	0.0635217	0.115566	-0.386453
LW3YPCA	LW2YA1A	10433039	0.0684828	0.121206	-0.386465
LW3YSDA	LW2YD1A	10121296	0.0757738	0.123911	-0.38666
LW3YMGA	LW0YVKA	10465207	0.0782987	0.130319	-0.386691
LW2YKCA	LW2YIGA	10441030	0.0764096	0.108728	-0.386805
LW1YTFA	LW0YGCA	10449227	0.0726981	0.124613	-0.387702
LW3YXDA	LW0YIBA	10478951	0.0746473	0.120482	-0.388014
LW1YS2A	LW0YPDA	10420394	0.0779896	0.130347	-0.388203
LW3YVBA	LW0YKFA	10414088	0.0726181	0.131536	-0.388655

LW0YKFA	LW0YIBA	10475586	0.078239	0.133575	-0.388864
LW2YIGA	LW0YDCA	10489220	0.0678761	0.117955	-0.388942
LW2YKCA	LW1YR2A	10438951	0.0807728	0.122229	-0.38902
LW3YHAA	LW0YGAA	10442202	0.0732764	0.124249	-0.389511
LW3YA1A	LW0YIBA	10498595	0.0759303	0.114789	-0.389531
LW3YVCA	LW3YMGA	10302424	0.0763292	0.126226	-0.389588
LW3YHAA	LW0YRBA	10326868	0.074143	0.123541	-0.389793
LW1YA2A	LW0YWCA	10447866	0.0673762	0.11194	-0.390064
LW2YQ1A	LW0YDCA	10493234	0.0652975	0.117604	-0.390133
LW2YA1A	LW0YVKA	10447356	0.0709748	0.125192	-0.390136
LW3YQAA	LW0YCBA	10159814	0.0735219	0.104713	-0.390363
LW2YIGA	LW1YA2A	10508834	0.0709942	0.118538	-0.390521
LW3YXDA	LW1YQBA	10463948	0.0705131	0.125885	-0.391075
LW2YQ1A	LW0YGCA	10478044	0.0651858	0.116961	-0.391109
LW3YPCA	LW0YCBA	10376197	0.0713844	0.116107	-0.391333
LW3YSDA	LW1YS2A	10137300	0.0764767	0.127597	-0.391346
LW0YIBA	LW0YGAA	10458444	0.0760888	0.123065	-0.391417
LW0YGCA	LW0YDCA	10453740	0.0689331	0.12638	-0.391985
LW3YVCA	LW0YKFA	10314728	0.070995	0.130001	-0.392095
LW3YSDA	LW3YKGA	10110002	0.0794328	0.126043	-0.392098
LW1YQBA	LW0YRBA	10328055	0.0725155	0.127096	-0.392219
LW3YVBA	LW2YIGA	10430081	0.0646543	0.113988	-0.392241
LW2YQ1A	LW2YKCA	10445459	0.0755476	0.110295	-0.392483
LW0YXBA	LW0YRBA	10340453	0.0730579	0.125841	-0.392725
LW2YIGA	LW1YQBA	10476410	0.0675252	0.115254	-0.392968
LW3YMGA	LW0YPDA	10396585	0.0757025	0.128493	-0.393078
LW0YPDA	LW0YIBA	10407016	0.0783874	0.130215	-0.393098
LW0YVKA	LW0YKFA	10477474	0.0725717	0.134919	-0.393142
LW0YKFA	LW0YGAA	10461101	0.0731794	0.127452	-0.393208
LW1YA2A	LW0YKFA	10492244	0.0710165	0.127127	-0.393272
LW3YVCA	LW1YA2A	10329423	0.0749563	0.131418	-0.393489
LW1YQBA	LW0YDCA	10456105	0.0706386	0.124933	-0.393589
LW2YD1A	LW0YPDA	10404096	0.0785411	0.130066	-0.393852
LW3YHAA	LW0YHCA	10471337	0.0727807	0.115916	-0.394067
LW3YSDA	LW0YXBA	10121418	0.069298	0.128298	-0.394206
LW3YMGA	LW1YFBA	10474201	0.0786267	0.124883	-0.394418
LW1YR2A	LW0YHCA	10502837	0.0718328	0.121635	-0.394428
LW0YVKA	LW0YNAA	10350352	0.0696046	0.13336	-0.394562
LW2YR2A	LW1YQBA	10466745	0.0709726	0.118181	-0.39497
LW0YNAA	LW0YHCA	10362897	0.0673003	0.116955	-0.395073
LW3YA1A	LW0YGAA	10483456	0.0601973	0.117723	-0.395133
LW3YSDA	LW0YPDA	10062184	0.0711491	0.131537	-0.395443
LW3YVCA	LW3YSDA	9970351	0.0695608	0.131399	-0.395678
LW1YTFA	LW0YGAA	10451545	0.0716308	0.126384	-0.395693
LW0YPDA	LW0YGCA	10389553	0.0708933	0.128164	-0.395711
LW1YR2A	LW0YPDA	10423078	0.0669394	0.126899	-0.395879

LW3YSDA	LW0YIBA	10124042	0.0737476	0.125799	-0.395924
LW3YKGA	LW0YPDA	10393078	0.0785318	0.129448	-0.396035
LW2YKCA	LW0YRBA	10293252	0.0810752	0.122717	-0.396131
LW1YTFA	LW0YPDA	10400025	0.073139	0.132937	-0.396356
LW2YEHA	LW0YPDA	10399943	0.0847559	0.126304	-0.39722
LW1YYAA	LW0YNAA	10319208	0.076182	0.131269	-0.39734
LW3YVCA	LW2YEHA	10305433	0.084354	0.124476	-0.397683
LW0YNAA	LW0YDCA	10347112	0.0671901	0.124275	-0.397685
LW2YA1A	LW0YGAA	10430518	0.0682305	0.128699	-0.397853
LW2YIGA	LW0YWCA	10449565	0.0626451	0.115855	-0.398221
LW2YEHA	LW0YKFA	10468375	0.0847196	0.131069	-0.398263
LW3YXDA	LW0YHCA	10491797	0.0657811	0.120095	-0.398563
LW0YVKA	LW0YBFA	10472797	0.0676026	0.119275	-0.399148
LW3YXDA	LW1YI2A	10489799	0.0649329	0.123095	-0.399154
LW0YVKA	LW0YIBA	10475575	0.0827221	0.136174	-0.399586
LW2YKCA	LW0YGCA	10405473	0.0789398	0.123459	-0.400375
LW3YJBA	LW0YBFA	10441131	0.066021	0.120471	-0.400411
LW3YHAA	LW0YDCA	10454423	0.0688056	0.122108	-0.40056
LW0YKFA	LW0YDCA	10473425	0.0708862	0.125443	-0.40064
LW0YHCA	LW0YCBA	10401298	0.0710436	0.115705	-0.401142
LW0YRBA	LW0YNAA	10221097	0.0695848	0.127545	-0.401238
LW0YXBA	LW0YDCA	10468889	0.0710644	0.125517	-0.401411
LW3YVBA	LW1YYAA	10380423	0.0766399	0.131703	-0.402353
LW3YVCA	LW1YTFA	10305320	0.0710373	0.131747	-0.402711
LW1YTFA	LW1YFBA	10476939	0.0737202	0.129418	-0.402882
LW0YRBA	LW0YGCA	10325564	0.070991	0.131567	-0.402989
LW3YSDA	LW2YEHA	10117185	0.0838237	0.124325	-0.403099
LW1YA2A	LW0YRBA	10359271	0.0739546	0.132454	-0.403113
LW1YR2A	LW0YIBA	10489616	0.0783607	0.127231	-0.403163
LW3YPCA	LW3YJBA	10431229	0.0625059	0.119031	-0.40363
LW3YJBA	LW2YR2A	10451289	0.0605875	0.119411	-0.403784
LW3YSDA	LW0YCBA	10044740	0.0764872	0.131173	-0.403874
LW3YXDA	LW3YSDA	10128773	0.0689799	0.129582	-0.40395
LW3YVBA	LW1YA2A	10428115	0.0695263	0.13082	-0.404069
LW0YGAA	LW0YDCA	10456052	0.0662435	0.127876	-0.40422
LW1YI2A	LW0YPDA	10417664	0.0678805	0.123011	-0.40423
LW3YRDA	LW1YI2A	10476946	0.0719514	0.119061	-0.404458
LW2YIGA	LW0YCBA	10406122	0.0690974	0.113905	-0.40447
LW1YQBA	LW1YFBA	10469135	0.0659166	0.128327	-0.404498
LW3YJBA	LW2YA1A	10415234	0.0660534	0.119035	-0.404984
LW1YI2A	LW0YBFA	10481872	0.0668838	0.125651	-0.405384
LW0YWCA	LW0YHCA	10444463	0.0619905	0.116425	-0.405393
LW3YKGA	LW0YNAA	10334668	0.0793942	0.13329	-0.405831
LW3YA1A	LW0YKFA	10500846	0.0687758	0.120524	-0.405987
LW0YRBA	LW0YGAA	10327902	0.0644705	0.128916	-0.40609
LW2YR2A	LW0YXBA	10479141	0.0725309	0.119855	-0.406228

LW3YVBA	LW2YD1A	10408935	0.0779999	0.132921	-0.406751
LW2YKCA	LW1YFBA	10433289	0.0773712	0.125141	-0.407023
LW1YR2A	LW0YDCA	10487556	0.0700612	0.130342	-0.407217
LW3YXDA	LW0YRBA	10347674	0.068743	0.131825	-0.407576
LW3YSDA	LW3YRDA	10115170	0.0752603	0.130231	-0.407812
LW3YVBA	LW3YSDA	10064818	0.0691113	0.133513	-0.407982
LW3YVCA	LW3YA1A	10336971	0.0716683	0.124946	-0.40886
LW2YR2A	LW1YI2A	10492292	0.0607515	0.121051	-0.408867
LW2YEHA	LW1YQBA	10450523	0.0835361	0.129443	-0.408881
LW1YS2A	LW0YWCA	10445356	0.0747516	0.112414	-0.40949
LW3YPCA	LW0YHCA	10474312	0.0673345	0.125516	-0.409564
LW2YR2A	LW2YQ1A	10503314	0.0629577	0.123787	-0.409567
LW3YSDA	LW1YQBA	10107982	0.0726258	0.135353	-0.409594
LW3YJBA	LW0YDCA	10440843	0.064012	0.119606	-0.409704
LW2YKCA	LW0YBFA	10421071	0.0752524	0.116523	-0.40973
LW3YSDA	LW0YKFA	10126622	0.0670386	0.131237	-0.409967
LW3YMGA	LW1YR2A	10479475	0.0777726	0.128159	-0.410071
LW2YQ1A	LW2YA1A	10467825	0.0663377	0.122231	-0.410277
LW1YYAA	LW0YKFA	10443597	0.0742965	0.137293	-0.410294
LW1YI2A	LW0YNAA	10358781	0.0685802	0.124271	-0.410938
LW3YPCA	LW1YA2A	10477558	0.0684495	0.126149	-0.41158
LW3YRDA	LW0YBFA	10462968	0.0703054	0.118276	-0.412079
LW3YSDA	LW2YEBA	10099873	0.0837721	0.127682	-0.412218
LW2YR2A	LW0YGAA	10466422	0.0645299	0.121424	-0.412296
LW0YXBA	LW0YVKA	10472645	0.0698186	0.138401	-0.412481
LW1YA2A	LW0YPDA	10424106	0.070567	0.133424	-0.412606
LW3YHAA	LW3YA1A	10482199	0.0671066	0.118997	-0.412666
LW3YSDA	LW0YGAA	10110334	0.0649321	0.130995	-0.412875
LW2YKCA	LW0YGAA	10408030	0.0756155	0.125377	-0.412906
LW3YKGA	LW0YXBA	10456900	0.0804975	0.139838	-0.413083
LW3YXDA	LW3YQAA	10248101	0.0639211	0.112413	-0.413134
LW2YEBA	LW0YXBA	10443061	0.0812621	0.134763	-0.413169
LW3YJBA	LW0YKFA	10445779	0.0744859	0.12066	-0.413445
LW3YQAA	LW0YWCA	10202304	0.0637829	0.11816	-0.414371
LW1YFBA	LW0YBFA	10481639	0.0655059	0.124687	-0.414573
LW3YA1A	LW2YR2A	10506158	0.0622318	0.123736	-0.414609
LW1YFBA	LW0YRBA	10353298	0.0646097	0.130917	-0.414742
LW2YEBA	LW0YKFA	10447995	0.0833005	0.135741	-0.414905
LW3YVBA	LW2YR2A	10419524	0.0655955	0.120475	-0.415014
LW2YR2A	LW0YCBA	10395724	0.0689925	0.117167	-0.415506
LW1YTFA	LW0YKFA	10468396	0.0720464	0.141902	-0.415547
LW0YHCA	LW0YDCA	10484128	0.0658943	0.124569	-0.415603
LW3YQAA	LW0YXBA	10241032	0.0703494	0.111672	-0.415733
LW3YA1A	LW0YXBA	10495585	0.0704299	0.123378	-0.415874
LW3YXDA	LW2YKCA	10428077	0.0747784	0.124578	-0.416061
LW3YXDA	LW0YWCA	10436253	0.0652242	0.11736	-0.41634

LW2YA1A	LW1YFBA	10455786	0.0637137	0.130902	-0.41638
LW3YJBA	LW0YGCA	10426751	0.0670516	0.121707	-0.416613
LW1YFBA	LW0YIBA	10483912	0.0721499	0.127592	-0.416867
LW1YA2A	LW0YVKA	10491346	0.0696866	0.132989	-0.416876
LW2YIGA	LW0YGAA	10476527	0.0672674	0.122886	-0.416938
LW1YFBA	LW0YKFA	10486116	0.069239	0.131539	-0.416998
LW3YQAA	LW2YR2A	10251123	0.063916	0.116743	-0.417114
LW3YQAA	LW3YJBA	10214446	0.0591852	0.115073	-0.41722
LW3YVBA	LW3YA1A	10436725	0.0719182	0.126438	-0.417222
LW3YVBA	LW0YBFA	10409638	0.0687649	0.124961	-0.41731
LW3YSDA	LW1YFBA	10133870	0.067537	0.13371	-0.417663
LW3YVCA	LW2YIGA	10330090	0.0652235	0.120572	-0.417665
LW2YIGA	LW0YNAA	10367654	0.065141	0.119516	-0.417696
LW3YMGA	LW1YA2A	10479768	0.0736061	0.128627	-0.417788
LW0YPDA	LW0YKFA	10408677	0.0716136	0.138404	-0.41782
LW3YPCA	LW2YKCA	10410554	0.0748326	0.119176	-0.417987
LW3YQAA	LW3YPCA	10231474	0.0628559	0.115158	-0.418141
LW2YD1A	LW1YR2A	10487161	0.0718882	0.127271	-0.418199
LW3YSDA	LW2YA1A	10096853	0.0645362	0.130895	-0.418445
LW1YYAA	LW1YQBA	10425632	0.0715181	0.134236	-0.418708
LW1YR2A	LW0YKFA	10491692	0.0682317	0.131441	-0.418874
LW1YQBA	LW0YHCA	10471642	0.0710615	0.124444	-0.418997
LW2YKCA	LW0YDCA	10420347	0.0753418	0.123969	-0.419083
LW3YVBA	LW2YQ1A	10433519	0.0690042	0.122987	-0.419103
LW3YVCA	LW2YD1A	10309383	0.0742623	0.132495	-0.419204
LW3YVCA	LW1YYAA	10281216	0.0728654	0.132545	-0.419408
LW0YPDA	LW0YGAA	10392223	0.0681552	0.133179	-0.419601
LW2YR2A	LW0YHCA	10494314	0.0619438	0.125341	-0.420076
LW2YQ1A	LW1YI2A	10505760	0.0607344	0.123562	-0.420192
LW0YXBA	LW0YWCA	10428920	0.0677829	0.115059	-0.42054
LW2YIGA	LW0YXBA	10489245	0.0703794	0.121238	-0.420581
LW2YR2A	LW0YDCA	10478991	0.0613813	0.122827	-0.421144
LW1YI2A	LW0YXBA	10481577	0.067676	0.125239	-0.42149
LW0YHCA	LW0YGCA	10469128	0.0685346	0.126569	-0.422051
LW2YIGA	LW0YKFA	10493848	0.0658029	0.119504	-0.422171
LW2YD1A	LW0YRBA	10340077	0.0770283	0.129244	-0.422242
LW3YRDA	LW0YVKA	10467308	0.0741131	0.138699	-0.422268
LW3YSDA	LW0YVKA	10124695	0.0673994	0.135372	-0.422647
LW3YPCA	LW3YHAA	10444540	0.0678254	0.12259	-0.422752
LW1YI2A	LW0YKFA	10486331	0.0683684	0.125952	-0.422961
LW1YTFA	LW0YVKA	10467909	0.0747703	0.142633	-0.423011
LW2YQ1A	LW1YA2A	10512725	0.0652986	0.12385	-0.423345
LW3YXDA	LW3YMGA	10468399	0.0724486	0.127393	-0.423496
LW2YEHA	LW0YGAA	10451002	0.0795019	0.12445	-0.423597
LW0YPDA	LW0YBFA	10404949	0.066477	0.125291	-0.424075
LW3YRDA	LW3YPCA	10453538	0.0719359	0.122796	-0.424197

LW2YA1A	LW0YHCA	10458541	0.0700353	0.128088	-0.424254
LW3YA1A	LW0YHCA	10511385	0.0659123	0.128547	-0.424414
LW0YWCA	LW0YGCA	10413846	0.0670775	0.11979	-0.424425
LW2YQ1A	LW0YWCA	10452805	0.0604126	0.120369	-0.425297
LW3YRDA	LW2YQ1A	10487511	0.0744344	0.120727	-0.42556
LW3YPCA	LW0YPDA	10394722	0.0653776	0.126067	-0.42558
LW2YIGA	LW0YHCA	10505080	0.0645531	0.127068	-0.425725
LW3YA1A	LW1YR2A	10514337	0.0717484	0.130389	-0.426043
LW3YVCA	LW0YVKA	10313087	0.069014	0.138342	-0.426341
LW3YQAA	LW0YGCA	10226753	0.0611877	0.113702	-0.426722
LW3YVBA	LW0YRBA	10281944	0.0652685	0.132175	-0.426832
LW2YD1A	LW2YA1A	10443115	0.0739256	0.128896	-0.427225
LW3YA1A	LW1YQBA	10482649	0.0716702	0.128172	-0.42742
LW3YVBA	LW2YEHA	10404191	0.0816253	0.13218	-0.427446
LW1YS2A	LW0YVKA	10488691	0.0716625	0.139457	-0.427839
LW3YXDA	LW0YPDA	10411771	0.0687285	0.134153	-0.427959
LW2YD1A	LW1YFBA	10481753	0.077681	0.132576	-0.428164
LW3YSDA	LW2YQ1A	10146232	0.0656474	0.12448	-0.428482
LW2YQ1A	LW1YR2A	10511399	0.064134	0.124685	-0.428585
LW3YRDA	LW2YR2A	10473301	0.0719369	0.120542	-0.429297
LW2YQ1A	LW0YNAA	10372058	0.0660107	0.123592	-0.430402
LW2YD1A	LW0YGCA	10453258	0.0736898	0.130516	-0.430553
LW3YVCA	LW2YEBA	10285578	0.0812505	0.132355	-0.431167
LW3YJBA	LW1YR2A	10459449	0.0688815	0.125775	-0.431529
LW3YJBA	LW1YA2A	10459918	0.0644553	0.12341	-0.43153
LW3YJBA	LW0YCBA	10358220	0.0694615	0.11899	-0.431652
LW3YJBA	LW0YHCA	10456411	0.065595	0.127809	-0.432167
LW3YVCA	LW0YHCA	10325384	0.0685661	0.127144	-0.432314
LW3YA1A	LW2YKCA	10447849	0.0743423	0.121367	-0.432373
LW0YWCA	LW0YPDA	10365302	0.0623145	0.116936	-0.432655
LW1YR2A	LW0YRBA	10358072	0.0673415	0.136631	-0.432859
LW3YQAA	LW1YS2A	10256506	0.0756719	0.114206	-0.432878
LW3YVCA	LW2YR2A	10320036	0.0656399	0.125149	-0.432984
LW3YQAA	LW2YIGA	10260561	0.0614933	0.119102	-0.432994
LW2YQ1A	LW0YGAA	10480459	0.0650726	0.126234	-0.433128
LW3YVBA	LW3YQAA	10183537	0.0651265	0.114832	-0.433742
LW3YPCA	LW0YDCA	10458946	0.0655762	0.131273	-0.433762
LW3YJBA	LW3YA1A	10467810	0.0598031	0.124908	-0.43402
LW3YA1A	LW0YGCA	10480512	0.0645019	0.128855	-0.434293
LW3YVBA	LW0YVKA	10413414	0.0690969	0.142096	-0.434412
LW3YVBA	LW0YDCA	10409051	0.0707996	0.135127	-0.434549
LW3YVCA	LW3YJBA	10282450	0.0641577	0.122798	-0.435114
LW3YMGA	LW0YGAA	10447895	0.0754951	0.132998	-0.435428
LW3YPCA	LW1YFBA	10471864	0.0649065	0.130372	-0.43634
LW1YI2A	LW1YA2A	10500097	0.068073	0.13243	-0.43655
LW0YIBA	LW0YBFA	10471245	0.0728843	0.124315	-0.437147

LW3YJBA	LW0YXBA	10440994	0.0692771	0.123195	-0.437184
LW2YEHA	LW1YFBA	10476501	0.0805445	0.128554	-0.437197
LW3YXDA	LW2YQ1A	10501033	0.0686008	0.129321	-0.437292
LW0YWCA	LW0YGAA	10416269	0.0652269	0.121523	-0.437544
LW3YJBA	LW0YWCA	10400938	0.0603505	0.123656	-0.437606
LW2YA1A	LW0YPDA	10378946	0.0646109	0.134382	-0.437678
LW3YVCA	LW2YQ1A	10334031	0.061683	0.124306	-0.437712
LW3YQAA	LW2YQ1A	10264132	0.0610527	0.119667	-0.437731
LW1YQBA	LW0YPDA	10390681	0.0689669	0.143614	-0.437981
LW0YVKA	LW0YGAA	10460206	0.0686447	0.136966	-0.438039
LW3YQAA	LW0YHCA	10255939	0.0619302	0.119802	-0.438132
LW1YFBA	LW0YDCA	10481302	0.0629075	0.134179	-0.43819
LW3YJBA	LW0YGAA	10428423	0.0654546	0.125798	-0.439317
LW3YA1A	LW0YWCA	10455892	0.0593192	0.122056	-0.439753
LW3YQAA	LW1YA2A	10258658	0.0673181	0.118923	-0.439998
LW3YMGA	LW0YGCA	10445648	0.0745667	0.133468	-0.441121
LW3YA1A	LW1YI2A	10509087	0.0657284	0.133262	-0.441389
LW3YSDA	LW0YGCA	10105725	0.0625606	0.136289	-0.441578
LW1YI2A	LW0YHCA	10496938	0.0596875	0.12879	-0.441791
LW3YRDA	LW0YHCA	10478339	0.0737283	0.125095	-0.441984
LW3YRDA	LW3YJBA	10436064	0.0757726	0.123144	-0.442073
LW1YQBA	LW0YVKA	10460111	0.0653568	0.143349	-0.442237
LW3YVBA	LW0YHCA	10425135	0.0673823	0.128326	-0.442452
LW2YEBA	LW1YQBA	10429787	0.0787022	0.136477	-0.442453
LW1YTFA	LW0YDCA	10464113	0.0738171	0.13587	-0.442508
LW3YSDA	LW1YR2A	10138644	0.0652401	0.138626	-0.442893
LW2YKCA	LW1YI2A	10433002	0.071585	0.12419	-0.442938
LW0YHCA	LW0YGAA	10471326	0.0659973	0.130052	-0.443669
LW2YA1A	LW1YI2A	10455751	0.0648286	0.133481	-0.443683
LW3YA1A	LW0YCBA	10412475	0.0738808	0.128525	-0.443799
LW3YPCA	LW3YMGA	10451270	0.0732262	0.126659	-0.444081
LW2YKCA	LW0YHCA	10436230	0.0778156	0.12398	-0.444425
LW2YA1A	LW1YTFA	10438819	0.07276	0.137308	-0.444611
LW3YXDA	LW3YJBA	10448768	0.0723525	0.130937	-0.444896
LW0YWCA	LW0YNAA	10307656	0.0625484	0.119428	-0.445408
LW3YRDA	LW2YA1A	10436959	0.0725579	0.133929	-0.44555
LW3YHAA	LW2YIGA	10475378	0.0676908	0.123402	-0.44556
LW3YQAA	LW1YR2A	10258856	0.0664692	0.120104	-0.445849
LW3YMGA	LW1YI2A	10474125	0.0734722	0.128057	-0.446
LW3YVBA	LW1YFBA	10421832	0.0676094	0.139549	-0.446056
LW2YEBA	LW1YFBA	10456094	0.0787111	0.130999	-0.446474
LW2YD1A	LW0YGAA	10455678	0.0728042	0.134706	-0.447894
LW3YSDA	LW1YI2A	10133004	0.0663274	0.133297	-0.447946
LW1YYAA	LW0YGAA	10426777	0.0755804	0.136583	-0.448128
LW1YI2A	LW0YDCA	10481821	0.0637516	0.134532	-0.448132
LW3YHAA	LW2YR2A	10464749	0.0713057	0.126585	-0.44831

LW2YD1A	LW1YI2A	10481810	0.0784509	0.131133	-0.448803
LW1YS2A	LW0YRBA	10355821	0.0750279	0.136681	-0.449119
LW2YD1A	LW0YDCA	10468175	0.0712683	0.13127	-0.449154
LW2YQ1A	LW0YHCA	10508465	0.0582919	0.130052	-0.449351
LW3YHAA	LW0YGCA	10438936	0.0709973	0.137034	-0.44944
LW3YXDA	LW3YRDA	10470402	0.0724026	0.134965	-0.449597
LW3YRDA	LW2YIGA	10483253	0.0738903	0.125217	-0.450028
LW3YSDA	LW0YBFA	10122141	0.0633873	0.130945	-0.450122
LW0YVKA	LW0YGCA	10458172	0.065531	0.137946	-0.450387
LW3YQAA	LW0YGAA	10228416	0.064777	0.120324	-0.450565
LW3YXDA	LW2YIGA	10496814	0.0644093	0.12958	-0.450766
LW0YWCA	LW0YCBA	10346162	0.0685493	0.119013	-0.450994
LW3YRDA	LW0YRBA	10334153	0.0742459	0.136024	-0.45102
LW2YR2A	LW0YIBA	10481622	0.0697501	0.123575	-0.45156
LW3YSDA	LW3YPCA	10112224	0.0643794	0.132613	-0.451582
LW3YVBA	LW3YJBA	10381677	0.0645989	0.126044	-0.451633
LW2YR2A	LW1YFBA	10491737	0.0632504	0.129549	-0.451721
LW3YPCA	LW2YEHA	10454070	0.0781168	0.123323	-0.452314
LW3YPCA	LW2YD1A	10458673	0.0734534	0.128758	-0.452793
LW3YPCA	LW0YVKA	10463434	0.0666199	0.132161	-0.453055
LW3YKGA	LW0YKFA	10461477	0.0759491	0.147544	-0.453226
LW3YSDA	LW3YA1A	10148043	0.0659132	0.13295	-0.453466
LW2YIGA	LW1YFBA	10502030	0.0623279	0.128577	-0.453482
LW2YQ1A	LW2YD1A	10492709	0.0718122	0.124506	-0.453723
LW2YQ1A	LW0YXBA	10492663	0.0669268	0.127913	-0.454384
LW2YEBA	LW0YRBA	10315577	0.0820143	0.132603	-0.454397
LW3YXDA	LW0YVKA	10480263	0.0661298	0.138508	-0.454917
LW2YQ1A	LW0YCBA	10410126	0.0664674	0.12484	-0.455348
LW3YA1A	LW2YD1A	10495224	0.0735263	0.1285	-0.456232
LW3YVCA	LW1YFBA	10321941	0.0666194	0.142447	-0.457136
LW0YKFA	LW0YHCA	10488578	0.0654181	0.129049	-0.457966
LW3YA1A	LW0YDCA	10495638	0.069101	0.137521	-0.458189
LW3YHAA	LW1YI2A	10467309	0.0737424	0.134495	-0.458714
LW3YXDA	LW1YTFA	10471492	0.0700068	0.139545	-0.458873
LW3YVBA	LW2YEBA	10383631	0.0793206	0.140022	-0.459111
LW3YSDA	LW0YRBA	9998853	0.066908	0.141735	-0.459277
LW3YQAA	LW2YA1A	10215436	0.0678587	0.124215	-0.459288
LW3YKGA	LW1YA2A	10475643	0.0753202	0.138355	-0.459309
LW2YR2A	LW2YKCA	10430295	0.0732122	0.124373	-0.459426
LW2YR2A	LW1YTFA	10474289	0.0656226	0.127874	-0.459596
LW3YJBA	LW3YHAA	10426992	0.0687101	0.125934	-0.460064
LW3YSDA	LW2YIGA	10142078	0.0616934	0.128916	-0.460203
LW3YRDA	LW3YQAA	10235564	0.06836	0.115795	-0.460209
LW3YPCA	LW0YIBA	10461139	0.0729777	0.130734	-0.460458
LW3YQAA	LW3YA1A	10266626	0.0610082	0.1236	-0.460562
LW3YMGA	LW0YBFA	10461161	0.0733658	0.129611	-0.460756

LW3YQAA	LW0YDCA	10240962	0.0688298	0.125869	-0.4623
LW2YEHA	LW1YR2A	10481886	0.0790921	0.133699	-0.462303
LW0YRBA	LW0YHCA	10355550	0.065168	0.134402	-0.46241
LW1YFBA	LW0YVKA	10485742	0.0673094	0.142468	-0.462609
LW1YFBA	LW0YPDA	10416865	0.0671153	0.143853	-0.464689
LW3YPCA	LW1YTFA	10454228	0.0679081	0.133779	-0.465018
LW1YYAA	LW1YA2A	10458506	0.0714117	0.13972	-0.46565
LW3YVCA	LW0YDCA	10309823	0.0680261	0.141869	-0.465723
LW3YMGA	LW3YA1A	10487644	0.0706337	0.129366	-0.465995
LW2YEHA	LW1YA2A	10482295	0.0764327	0.13404	-0.466058
LW0YIBA	LW0YHCA	10486564	0.0714514	0.128644	-0.466518
LW1YYAA	LW0YGCA	10423825	0.070351	0.138003	-0.466542
LW1YI2A	LW0YIBA	10483804	0.0737227	0.133465	-0.466843
LW2YEHA	LW0YRBA	10335159	0.0826184	0.134993	-0.466854
LW3YJBA	LW0YIBA	10442882	0.0689934	0.124641	-0.467464
LW3YMGA	LW0YRBA	10332259	0.0729781	0.138056	-0.467487
LW3YA1A	LW1YTFA	10491195	0.0709217	0.134931	-0.46833
LW2YIGA	LW0YVKA	10493424	0.0634174	0.129634	-0.46873
LW1YTFA	LW0YBFA	10464408	0.0688825	0.134081	-0.468785
LW3YQAA	LW0YNAA	10121497	0.0617967	0.12022	-0.469238
LW1YQBA	LW0YWCA	10415944	0.0693237	0.127958	-0.469402
LW3YA1A	LW2YQ1A	10520362	0.0611641	0.135491	-0.469621
LW3YXBA	LW3YQAA	9924183	0.0565185	0.11768	-0.469784
LW1YYAA	LW0YRBA	10310618	0.07415	0.139744	-0.469957
LW1YYAA	LW1YR2A	10457372	0.0719748	0.140368	-0.47029
LW2YIGA	LW2YD1A	10488651	0.0711292	0.127115	-0.470533
LW2YA1A	LW0YWCA	10402903	0.0639112	0.128591	-0.470664
LW3YJBA	LW2YKCA	10392957	0.0748659	0.125811	-0.472153
LW3YJBA	LW0YNAA	10319271	0.0674266	0.131692	-0.472234
LW3YQAA	LW0YBFA	10239982	0.0636948	0.127368	-0.472429
LW3YXBA	LW3YPCA	10133838	0.0587024	0.114796	-0.472931
LW0YPDA	LW0YDCA	10403901	0.0633766	0.140589	-0.472963
LW3YA1A	LW0YRBA	10366475	0.0613359	0.136774	-0.473331
LW1YI2A	LW0YRBA	10352521	0.0663163	0.141229	-0.474055
LW2YIGA	LW0YIBA	10491723	0.0725049	0.12904	-0.474056
LW2YIGA	LW1YTFA	10484469	0.0675377	0.131103	-0.474293
LW2YKCA	LW0YWCA	10380674	0.0706574	0.120604	-0.475711
LW2YD1A	LW0YBFA	10468145	0.0729437	0.132924	-0.475946
LW3YJBA	LW1YFBA	10453662	0.0619661	0.132193	-0.475951
LW0YWCA	LW0YRBA	10301471	0.0673118	0.131427	-0.476119
LW2YR2A	LW0YKFA	10483687	0.0689288	0.134326	-0.477114
LW3YXDA	LW2YEHA	10471000	0.0768873	0.134831	-0.477241
LW3YVCA	LW3YQAA	10086009	0.0654323	0.124802	-0.477324
LW2YEBA	LW1YR2A	10461663	0.077592	0.137637	-0.477366
LW1YTFA	LW0YRBA	10335523	0.0659915	0.142005	-0.477613
LW1YYAA	LW1YFBA	10451786	0.0737808	0.143417	-0.478564

LW0YWCA	LW0YDCA	10428263	0.0595161	0.128681	-0.47876
LW2YIGA	LW0YRBA	10360203	0.0645309	0.136073	-0.478864
LW3YQAA	LW1YI2A	10253930	0.0593507	0.126012	-0.479059
LW3YQAA	LW0YIBA	10243186	0.0659039	0.117717	-0.47922
LW2YEBA	LW1YA2A	10463684	0.0764408	0.13839	-0.479365
LW2YEHA	LW0YGCA	10448026	0.0774721	0.136662	-0.480288
LW0YRBA	LW0YIBA	10341875	0.0733426	0.141788	-0.480383
LW2YEBA	LW0YGAA	10431097	0.0783253	0.138611	-0.480472
LW3YMGA	LW2YR2A	10471553	0.0701436	0.129931	-0.480909
LW3YJBA	LW1YTFA	10435963	0.063672	0.129632	-0.481706
LW3YXDA	LW1YFBA	10488551	0.0645396	0.147171	-0.482196
LW2YQ1A	LW1YTFA	10488567	0.067472	0.133698	-0.482291
LW1YTFA	LW1YI2A	10476739	0.0681327	0.13891	-0.483101
LW3YQAA	LW2YKCA	10193801	0.067333	0.1166	-0.483403
LW3YXDA	LW2YD1A	10475456	0.068972	0.140152	-0.483454
LW3YA1A	LW0YPDA	10431359	0.0627577	0.13712	-0.483491
LW3YA1A	LW0YVKA	10499727	0.0631513	0.136349	-0.483632
LW0YPDA	LW0YHCA	10419838	0.066127	0.13715	-0.484501
LW2YIGA	LW2YEBA	10464661	0.0711863	0.123989	-0.485435
LW3YHAA	LW0YWCA	10414383	0.066096	0.126238	-0.485913
LW2YQ1A	LW0YPDA	10428429	0.063533	0.135246	-0.485927
LW3YVBA	LW3YKGA	10397521	0.0685471	0.147186	-0.48663
LW2YD1A	LW0YHCA	10483389	0.0685197	0.131524	-0.487019
LW3YSDA	LW0YDCA	10121301	0.0642942	0.145654	-0.487065
LW3YKGA	LW0YRBA	10328824	0.0774082	0.143872	-0.487257
LW1YI2A	LW0YVKA	10486403	0.0652542	0.140313	-0.487674
LW3YMGA	LW2YQ1A	10485140	0.0692513	0.130956	-0.487684
LW3YMGA	LW3YJBA	10433086	0.0708651	0.12962	-0.487818
LW2YIGA	LW0YPDA	10424545	0.0608189	0.13395	-0.489403
LW2YQ1A	LW1YFBA	10505563	0.065432	0.139083	-0.489773
LW3YMGA	LW0YDCA	10460664	0.0743303	0.142449	-0.490039
LW1YR2A	LW0YVKA	10491096	0.0612454	0.146075	-0.490048
LW2YQ1A	LW2YEHA	10488089	0.0750149	0.126628	-0.490256
LW3YRDA	LW3YA1A	10489731	0.0681748	0.135088	-0.490275
LW1YI2A	LW1YFBA	10494243	0.0625611	0.142768	-0.491167
LW2YEHA	LW2YA1A	10437243	0.0773136	0.138574	-0.492755
LW3YMGA	LW2YIGA	10481397	0.0706075	0.131962	-0.492912
LW2YA1A	LW1YYAA	10413556	0.0741421	0.145262	-0.493057
LW0YVKA	LW0YDCA	10472489	0.0663517	0.145928	-0.493203
LW3YMGA	LW0YWCA	10420748	0.0685924	0.125633	-0.493309
LW3YVCA	LW3YKGA	10297330	0.0714587	0.148658	-0.494214
LW2YR2A	LW2YD1A	10478599	0.0668281	0.131604	-0.49543
LW0YRBA	LW0YDCA	10339722	0.0637533	0.148986	-0.49614
LW2YR2A	LW0YRBA	10350143	0.0652902	0.141249	-0.496333
LW3YHAA	LW2YQ1A	10478696	0.0652373	0.134663	-0.497523
LW3YQAA	LW0YKFA	10245166	0.0663191	0.127169	-0.497832

LW3YJBA	LW0YRBA	10312997	0.0651125	0.139266	-0.498275
LW1YYAA	LW1YI2A	10451771	0.0729606	0.140515	-0.498603
LW3YSDA	LW2YR2A	10130619	0.0623561	0.138723	-0.498819
LW3YA1A	LW2YEBA	10470676	0.074559	0.132888	-0.50082
LW3YQAA	LW3YMGA	10233077	0.0695945	0.123749	-0.5011
LW3YXBA	LW0YWCA	10104590	0.0569383	0.12294	-0.501353
LW3YJBA	LW0YPDA	10377008	0.0667685	0.138412	-0.502431
LW2YEBA	LW0YDCA	10443386	0.0743081	0.13901	-0.502759
LW3YKGA	LW1YQBA	10444094	0.073868	0.155174	-0.503515
LW3YPCA	LW2YEBA	10433381	0.074668	0.134719	-0.504589
LW2YEBA	LW2YA1A	10417087	0.074725	0.141203	-0.504631
LW2YIGA	LW2YEHA	10483999	0.0735919	0.128469	-0.505009
LW3YKGA	LW0YGAA	10444276	0.0723403	0.147318	-0.505611
LW3YXBA	LW0YGCA	10128099	0.0562579	0.117828	-0.506638
LW3YA1A	LW2YEHA	10490133	0.0731095	0.132683	-0.508362
LW1YTFA	LW0YHCA	10478907	0.0638583	0.139133	-0.509157
LW3YXDA	LW1YYAA	10446570	0.0722878	0.14842	-0.509243
LW2YD1A	LW0YWCA	10428291	0.0706914	0.130171	-0.509655
LW3YSDA	LW3YJBA	10094389	0.061798	0.138952	-0.509974
LW3YRDA	LW0YWCA	10422797	0.0695143	0.13099	-0.510105
LW2YEBA	LW0YGCA	10427661	0.0758292	0.144031	-0.510159
LW3YSDA	LW0YHCA	10136286	0.0632416	0.142761	-0.510713
LW3YXBA	LW1YS2A	10158678	0.0677633	0.11662	-0.511069
LW3YKGA	LW0YGCA	10442216	0.0745593	0.149526	-0.512399
LW3YKGA	LW1YR2A	10475252	0.0731728	0.150301	-0.5154
LW3YA1A	LW1YFBA	10507961	0.0594727	0.145439	-0.517959
LW3YJBA	LW0YVKA	10445495	0.0619795	0.139004	-0.519395
LW2YQ1A	LW0YVKA	10496728	0.0643607	0.142653	-0.520893
LW3YJBA	LW2YD1A	10440329	0.0721122	0.137883	-0.522188
LW2YEHA	LW1YI2A	10476477	0.0758587	0.139354	-0.522534
LW3YXBA	LW0YNAA	10028921	0.0620173	0.121567	-0.523827
LW3YA1A	LW1YYAA	10466176	0.0680048	0.142187	-0.524517
LW3YKGA	LW0YDCA	10456403	0.0708494	0.148374	-0.526505
LW3YSDA	LW0YWCA	10083175	0.0627327	0.138786	-0.527375
LW3YXBA	LW3YA1A	10169330	0.0600349	0.126728	-0.527958
LW0YWCA	LW0YKFA	10432783	0.0683947	0.138919	-0.528376
LW3YXBA	LW1YI2A	10154313	0.0575384	0.125108	-0.529118
LW2YQ1A	LW2YEBA	10468000	0.0741494	0.136157	-0.529508
LW0YWCA	LW0YIBA	10430700	0.0673042	0.133529	-0.530547
LW2YR2A	LW0YPDA	10414232	0.0630731	0.145523	-0.531993
LW3YXDA	LW2YEBA	10450585	0.0711373	0.146474	-0.533007
LW1YYAA	LW0YDCA	10438445	0.0716716	0.152002	-0.533264
LW1YTFA	LW0YWCA	10423952	0.0664018	0.138129	-0.533537
LW2YEHA	LW0YDCA	10463108	0.0754453	0.145932	-0.534489
LW3YMGA	LW0YHCA	10475909	0.0681156	0.142089	-0.534668
LW2YQ1A	LW0YIBA	10494847	0.0657007	0.140621	-0.536414

LW3YXBA	LW1YQBA	10130027	0.0638999	0.124998	-0.536985
LW3YXBA	LW0YGAA	10132010	0.0593965	0.125503	-0.538026
LW3YPCA	LW1YYAA	10428631	0.0678053	0.146464	-0.538919
LW3YKGA	LW2YA1A	10430638	0.0709006	0.15312	-0.538982
LW3YQAA	LW0YRBA	10115571	0.0593494	0.137023	-0.539127
LW2YEBA	LW0YBFA	10443145	0.0715805	0.14015	-0.539224
LW3YXBA	LW3YVCA	9991806	0.0591306	0.124397	-0.540545
LW3YQAA	LW3YHAA	10225740	0.0624353	0.132003	-0.540773
LW2YEHA	LW0YBFA	10463067	0.0729158	0.140918	-0.544479
LW3YPCA	LW3YKGA	10447062	0.0745289	0.14953	-0.545026
LW1YYAA	LW0YHCA	10454299	0.0677452	0.144927	-0.54559
LW2YR2A	LW1YYAA	10449166	0.0679796	0.14424	-0.546285
LW0YVKA	LW0YHCA	10487870	0.0567701	0.14593	-0.546375
LW1YFBA	LW0YWCA	10441241	0.062944	0.143973	-0.546689
LW3YJBA	LW1YYAA	10411209	0.0638683	0.139925	-0.546742
LW3YXBA	LW0YBFA	10143711	0.0607645	0.131099	-0.547884
LW3YQAA	LW2YEHA	10234993	0.0705958	0.126717	-0.549121
LW2YEHA	LW0YHCA	10478628	0.0708933	0.139174	-0.549134
LW3YXBA	LW3YVBA	10085973	0.060998	0.126369	-0.549143
LW3YXDA	LW3YKGA	10463939	0.0709968	0.15702	-0.554383
LW3YQAA	LW1YTFA	10235363	0.0629377	0.136702	-0.554789
LW3YKGA	LW2YIGA	10477505	0.0719108	0.145553	-0.555542
LW3YKGA	LW1YI2A	10470489	0.0666423	0.149562	-0.55734
LW3YXBA	LW1YR2A	10160380	0.0597881	0.12962	-0.558395
LW2YR2A	LW0YVKA	10482699	0.0598528	0.14961	-0.560519
LW3YXBA	LW2YA1A	10118064	0.0589477	0.130309	-0.560806
LW3YJBA	LW2YEHA	10435567	0.0744876	0.140702	-0.563888
LW1YYAA	LW0YBFA	10438907	0.0653758	0.150239	-0.565296
LW3YXBA	LW2YQ1A	10166948	0.0574994	0.133955	-0.566109
LW2YEBA	LW0YHCA	10459017	0.0694191	0.143506	-0.566789
LW2YEBA	LW1YI2A	10455598	0.0749913	0.150826	-0.569835
LW0YWCA	LW0YVKA	10432036	0.0620147	0.146063	-0.571499
LW3YQAA	LW0YPDA	10177247	0.0564394	0.140032	-0.573005
LW3YXBA	LW3YJBA	10115421	0.0571883	0.136068	-0.574618
LW3YKGA	LW2YQ1A	10480825	0.0681383	0.149088	-0.575342
LW3YSDA	LW3YQAA	9902039	0.0598067	0.143579	-0.577415
LW3YJBA	LW2YEBA	10415375	0.0710569	0.143509	-0.57853
LW3YQAA	LW1YYAA	10211050	0.0632899	0.137674	-0.579116
LW3YKGA	LW3YA1A	10483172	0.065993	0.152256	-0.580082
LW3YXBA	LW0YKFA	10147344	0.061325	0.131271	-0.582435
LW3YXBA	LW1YA2A	10162414	0.0595961	0.134335	-0.583428
LW3YKGA	LW2YR2A	10467175	0.06755	0.150979	-0.584212
LW3YQAA	LW1YFBA	10252042	0.0601336	0.146253	-0.584489
LW3YKGA	LW0YBFA	10456212	0.0643466	0.152677	-0.585078
LW2YQ1A	LW1YYAA	10463030	0.0651128	0.151701	-0.585944
LW3YQAA	LW2YD1A	10239517	0.0651217	0.140416	-0.590156

LW3YXBA	LW2YIGA	10162059	0.055975	0.138674	-0.592335
LW2YR2A	LW2YEHA	10473085	0.0740606	0.14959	-0.59333
LW2YEHA	LW0YWCA	10423097	0.0704647	0.142189	-0.600652
LW3YKGA	LW0YHCA	10471897	0.0701042	0.157502	-0.602688
LW2YR2A	LW2YEBA	10452865	0.0686936	0.150454	-0.603735
LW2YIGA	LW1YYAA	10459071	0.0679854	0.157591	-0.61026
LW3YXBA	LW0YCBA	10064709	0.0597742	0.133343	-0.612329
LW3YXBA	LW0YXBA	10142448	0.0606558	0.136866	-0.613235
LW3YKGA	LW3YJBA	10429550	0.0681807	0.156013	-0.616728
LW3YXBA	LW3YMGA	10134965	0.0633562	0.133866	-0.619599
LW2YEBA	LW0YWCA	10403073	0.0682612	0.146511	-0.62026
LW3YXBA	LW0YDCA	10142101	0.0554791	0.140801	-0.620266
LW1YYAA	LW0YWCA	10398818	0.0638356	0.151781	-0.621568
LW3YXBA	LW3YHAA	10128974	0.0600029	0.136156	-0.621869
LW3YXBA	LW0YIBA	10144377	0.0644961	0.136699	-0.628857
LW3YXBA	LW0YHCA	10156999	0.0566076	0.146305	-0.632391
LW3YXBA	LW0YRBA	10019294	0.0554934	0.144437	-0.640858
LW3YXBA	LW3YSDA	9811921	0.0568507	0.144378	-0.643675
LW3YQAA	LW0YVKA	10244242	0.0589461	0.155389	-0.643797
LW3YKGA	LW0YWCA	10416288	0.0636716	0.156544	-0.651652
LW3YQAA	LW2YEBA	10214743	0.0652037	0.146878	-0.652078
LW3YXBA	LW2YKCA	10096810	0.0637292	0.139675	-0.659332
LW3YXBA	LW1YTFA	10138604	0.0614159	0.1466	-0.664411
LW3YXBA	LW3YRDA	10135270	0.0603517	0.144795	-0.67573
LW3YXBA	LW1YFBA	10154155	0.0568883	0.154349	-0.690982
LW3YXBA	LW2YD1A	10141580	0.0588512	0.145964	-0.691856
LW3YXBA	LW0YPDA	10081214	0.0553283	0.152931	-0.697105
LW3YXBA	LW0YVKA	10145006	0.0544248	0.153066	-0.705326
LW3YQAA	LW3YKGA	10228060	0.0628357	0.164034	-0.712144
LW3YXBA	LW1YYAA	10114087	0.0576132	0.153671	-0.730421
LW3YXBA	LW2YEHA	10136828	0.0653117	0.150364	-0.737341
LW3YXBA	LW2YEBA	10118289	0.0640366	0.158439	-0.774841
LW3YXBA	LW3YKGA	10128876	0.0577875	0.17191	-0.831061
